# Rapid formation of human immunodeficiency virus-like particles

**DOI:** 10.1073/pnas.2008156117

**Published:** 2020-08-17

**Authors:** Joanna Bednarska, Annegret Pelchen-Matthews, Pavel Novak, Jemima J. Burden, Peter A. Summers, Marina K. Kuimova, Yuri Korchev, Mark Marsh, Andrew Shevchuk

**Affiliations:** ^a^Department of Medicine, Imperial College London, W12 0NN London, United Kingdom;; ^b^Medical Research Council Laboratory for Molecular Cell Biology, University College London, WC1E 6BT London, United Kingdom;; ^c^Functional Low-Dimensional Structures Laboratory, National University of Science and Technology “MISIS”, 119991 Moscow, Russian Federation;; ^d^Molecular Sciences Research Hub, Department of Chemistry, Imperial College London, W12 0BZ London, United Kingdom;; ^e^Nano Life Science Institute, Kanazawa University, 920-1192 Kanazawa, Japan

**Keywords:** HIV, assembly, SICM

## Abstract

Understanding the molecular mechanisms involved in the assembly of viruses is essential for discerning how viruses transmit from cell to cell and host to host. Although molecular aspects of assembly have been studied for many viruses, we still have little information about these events in real time. Enveloped viruses such as HIV that assemble at, and bud from, the plasma membrane have been studied in some detail using live cell fluorescence imaging techniques; however, these approaches provide little information about the real-time morphological changes that take place as viral components come together to form individual virus particles. Here we used correlative scanning ion conductance microscopy and fluorescence confocal microscopy to measure the topological changes, together with the recruitment of fluorescently labeled viral proteins such as Gag and Vpr, during the assembly and release of individual HIV virus-like particles (VLPs) from the top, nonadherent surfaces of living cells. We show that 1) labeling of viral proteins with green fluorescent protein affects particle formation, 2) the kinetics of particle assembly on different plasma membrane domains can vary, possibly as a consequence of differences in membrane biophysical properties, and 3) VLPs budding from the top, unimpeded surface of cells can reach full size in 20 s and disappear from the budding site in 0.5 to 3 min from the moment curvature is initially detected, significantly faster than has been previously reported.

## Significance

Currently, our knowledge of individual virus particle assembly kinetics is based solely on studies of the dynamics of fluorescent puncta corresponding to the viral structural protein called Gag labeled with fluorescent proteins (e.g., green fluorescent protein [GFP]) observed by fluorescence microscopy in membranes of cells. However, GFP tagging affects virus particle assembly and release. We directly measured topological changes during HIV-like particle assembly and found that they can reach full size in 20 s and release in 0.5 to 3 min. Compared to previous estimates of ∼8-min assembly time and 30- to 60-min release time, this is more than 10 times faster. In our opinion this is a highly important discovery that challenges current views on virus replication mechanisms.

Our current knowledge of individual retrovirus assembly kinetics is based solely on studies of the dynamics of fluorescent puncta observed by total internal reflection fluorescence microscopy (TIRFM) of the basal membrane of adherent cells expressing the viral polyprotein Gag fused to fluorescent proteins, for example green fluorescent protein (GFP) (1–3). Gag is the main structural protein of retroviruses and its expression alone is sufficient to drive the formation of virus-like particles (VLPs) (4). In TIRFM, the depth of field is ∼200 nm above the glass/surface interface. This minimizes out-of-focus, “background” fluorescence from within cells and provides images of sufficient contrast to clearly detect diffraction-limited fluorescent spots, representing putative individual VLPs on the basal surface of the cell. Whether the narrow spacing between the cell membrane and the glass hinders the formation and release of 120-nm-diameter virus particles remains unclear. The assumption that the dynamics of GFP-labeled Gag spots can serve as a model for the formation of virus particles with unmodified Gag is based on the fact that many of these spots colocalize with budding structures by correlative light and electron microscopy (CLEM) images (5). However, this latter study also reports that many of the observed GFP spots do not correspond to membrane-associated buds or VLPs visible by EM. Thus, the degree to which GFP (attached to Gag) affects VLP assembly and morphology remains unclear, as published results vary (5, 6). To attempt to avoid potential artifacts of GFP tagging, unlabeled Gag is usually cotransfected with Gag-GFP in ratios ranging from 1:1 to 1:10. Consequently, a wide range of stoichiometries of labeled and unlabeled Gag is likely at the single-cell level, resulting in heterogeneous Gag-GFP and unlabeled Gag content within VLPs (7).

Here we used high-speed (HS), correlative scanning ion conductance microscopy and fluorescence confocal microscopy (SICM-FCM) live-cell imaging to characterize and compare the formation of HIV VLPs on the top surfaces of cells, as opposed to the bottom surface in contact with a glass coverslip, typically studied by TIRFM. The observations were carried out on Jurkat, HeLa, Cos-7, and HEK293T cells expressing labeled and nonlabeled HIV Gag. SICM is a noncontact scanning probe microscopy technique that can image and measure the three-dimensional morphology of cells in physiological buffers with high spatial resolution (8, 9). Correlative SICM-FCM, similar to CLEM, produces pairs of surface and fluorescence images that can be superimposed to establish correlation. However, unlike CLEM, SICM-FCM is a live imaging technique that can follow the morphological changes of processes taking place at the cell surface, such as endocytosis and the uptake of viruses and nanoparticles (10–12). Here we show that the assembly and release of VLPs from the top, unimpeded surface of cells can be 20 times faster than that reported for VLP budding from plasma membrane (PM) in contact with a glass substrate by TIRFM. Also, the topological changes associated with VLP budding vary significantly depending on the cell model used and the viral protein that is labeled.

## Results

### VLP Formation in Jurkat Cells.

**Fig. 1.**
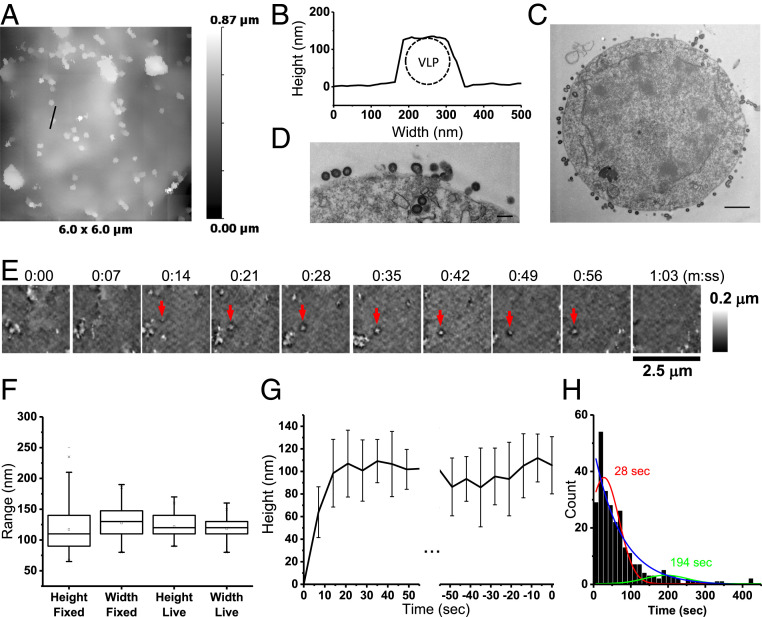
HIV VLP formation on Jurkat cells transfected with H2B-mPlum/Gag. (*A*) SICM image of VLPs on a cell fixed at 14 h posttransfection. (*B*) Cross-section profile of a membrane protrusion (black line) corresponding to a typical putative Gag VLP from *A*; the dashed circle represents a 125-nm-diameter VLP placed in the profile for reference. (*C* and *D*) TEM images of cells fixed at 19 h posttransfection showing VLPs. (*E*) HS-SICM time-lapse images showing the formation and disappearance of a VLP (red arrows) recorded starting from 8 h posttransfection. Images acquired at 8.6 frames per minute. (*F*) VLP bud heights and diameters (widths) measured in SICM topographical images. (*G*) Average VLP height change during assembly (*n* = 32, aligned to nucleation, *Left*) and prior to release (*n* = 42, aligned to disappearance, *Right*). (*H*) Distribution of VLP lifetimes. (TEM scale bars: 1 μm in *C* and 200 nm in *D*.)

We imaged Jurkat-TAg cells transfected with pH2B-mPlum/Gag (7) using a recently developed HS-SICM system. Cells expressing unlabeled Gag were identified through the expression of a fluorescently labeled histone. SICM topographical images of transfected cells fixed at 14 h posttransfection revealed PM protrusions that by size and shape could be identified as budding VLPs (Fig. 1*A* and *B*). Transmission electron microscopy (TEM) images confirmed the formation of VLPs with HIV-like appearance and sizes similar to those observed by SICM (Fig. 1*C* and *D*). SICM and TEM images of nontransfected Jurkat cells indicated that these cells have a smooth surface decorated with sparse microvilli (∼400 nm in diameter and up to several micrometers long) and do not have PM protrusions that could be topographically confused with VLPs or VLP buds (*SI Appendix*, Fig. S1*A*). Jurkat cells expressing H2B-mPlum/Gag formed VLPs at high efficiency, with VLPs found on 24 out of 30 imaged cells fixed between 14 and 24 h posttransfection. SICM images of cells fixed at 24 h indicated that the number of VLPs on the PM increased with time (*SI Appendix*, Fig. S1*B*). By immunostaining, we confirmed the absence of tetherin and TIM-1 (T-cell immunoglobulin and mucin domain 1) that could anchor released VLPs to the PM (*SI Appendix*, Fig. S1 *C* and *D*) (13, 14). It has also been previously demonstrated that Jurkat cells do not express tetherin and TIM-1 proteins (15, 16). Protease treatment of live cells with 100 μg/mL *Bacillus licheniformis* Subtilisin A resulted in a rapid 72 ± 21% reduction (two independent experiments) in the number of surface-bound VLPs (*SI Appendix*, Fig. S1 *E* and *F*), indicating that most of the observed VLP-like structures had completed scission but remained attached to the PM either by tetherin or TIM-1 present on the surface of cells at undetectable levels, or perhaps through alternative, as-yet-undefined, protein links.

We then followed individual Gag-driven VLP formation and release events in live cells at scan rates of 8.6 to 4.3 frames per minute (7 to 14 s per frame) (Fig. 1*E*). Comparison of PM protrusions in SICM topographical images of fixed and live cells indicated similar heights (median = 117 nm, interquartile range [IQR] = 140 to 90, *n* = 100, fixed and 122 nm, IQR = 140 to 110, *n* = 181, live) and diameters (127 nm, IQR = 147 to 110, *n* = 100, fixed and 118 nm, IQR = 130 to 110, *n* = 181, live) (Fig. 1*F*). VLP assembly was characterized by a progressive increase in the measured height of the protrusions which, on average, reached a maximum in 21 s (Fig. 1*G*, *Left*). The abrupt disappearance of the entire structure was interpreted as the release of VLP from the cell membrane (Fig. 1*G*, *Right*). We tracked a total of 267 individual VLPs (48 cells, 10 experiments) from the moment of initial membrane curvature to the complete disappearance of the topographically detected protrusions. The analysis of VLP lifetimes indicated a bimodal distribution with peaks at 28 and 194 s (Fig. 1*H*). A third population of protrusions, with possibly even longer lifetimes, could not be tracked as they drifted in and out of the area of observation; these are likely to represent VLPs that failed to complete scission or remained membrane-tethered after assembly was completed. Tracking VLP formation in live cells in the presence of 100 μg/mL Subtilisin A resulted in an average lifetime of 42 s (median, IQR = 48 to 24, *n* = 20, six cells, one experiment). This is longer than the average lifetime of short-lived VLPs on untreated cells, possibly due to a detrimental effect of the protease on live cells that often resulted in cells detaching from the substrate.

**Fig. 2.**
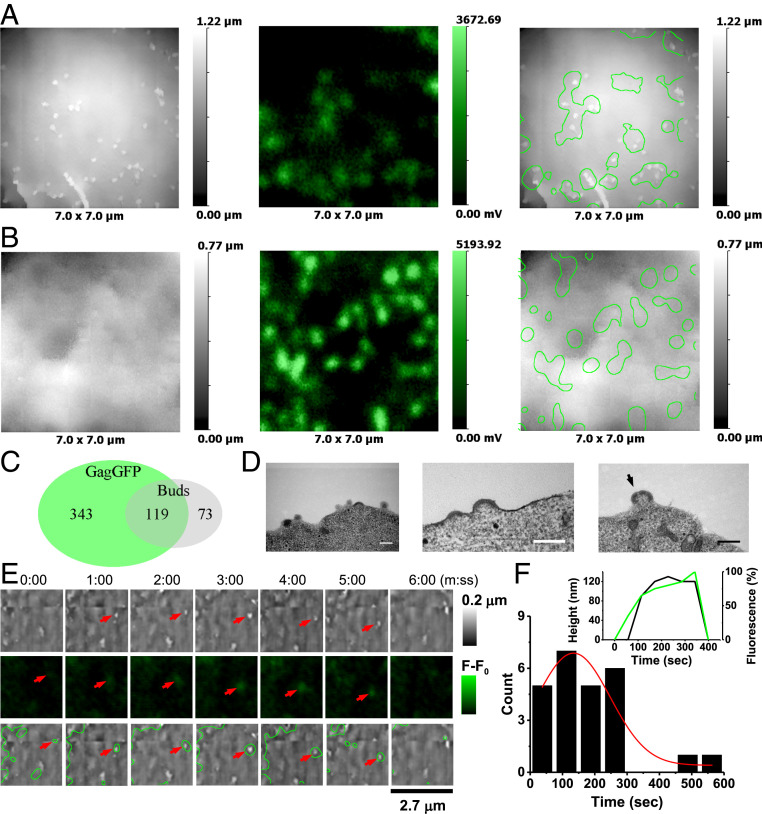
HIV VLP formation in Jurkat cells transfected with 1:1 Gag-GFP and SynGP. (*A* and *B*) Correlative SICM-FCM images of fixed cells showing examples of correlation between Gag-GFP fluorescence and VLP buds: SICM topographical image (*Left*), confocal fluorescence image (*Middle*), and overlay (*Right*). (*C*) Euler diagram showing the relation in sets of Gag-GFP fluorescence spots and topographically detected buds. (*D*) TEM images of VLP buds; the black arrow points at a discontinuity in the Gag layer. (Scale bars: 200 nm.) (*E*) SICM-FCM time-lapse images showing the formation and disappearance of a VLP (red arrows). Images acquired at one frame per minute. (*F*) Distribution of lifetimes of Gag-GFP–positive buds. (*Inset*) Bud height (black line) and fluorescence intensity traces (green line) for the VLP in *E*.

In order to confirm that the observed membrane protrusions represent VLPs formed by Gag, and to compare the dynamics of VLPs formed by labeled and nonlabeled Gag, we imaged Jurkat cells transfected with 1:1 Gag-GFP:SynGP by correlative SICM-FCM. SynGP is a codon-optimized synthetic Gag-Pol that can form proteolytically matured HIV VLPs (17). SICM-FCM images revealed numerous membrane protrusions (Fig. 2*A*, *Left*) and distinct fluorescence spots of Gag-GFP (Fig. 2*A*, *Middle*). Analysis of superimposed fluorescence and topographical images (Fig. 2*A*, *Right*) of Gag-GFP–positive cells fixed at 18 and 24 h posttransfection (16 and 14 cells, respectively) indicated that only five cells produced Gag-GFP fluorescence spots that correlated with topographically detected VLP buds (Fig. 2*A*). The majority, 17 cells, produced Gag-GFP fluorescence spots that correlated with flat or lightly curved membrane areas (Fig. 2*B*). In these 22 cells we identified a total of 462 Gag-GFP fluorescence spots and 192 topographically detected VLP buds, of which 119 overlapped (i.e., 26% Gag-GFP fluorescence spots correlated with budding structures, and 62% of the topographically detected buds had associated fluorescence). Collectively, these data are presented in the form of a Euler diagram in Fig. 2*C*. We did not observe an increase in the number of Gag-GFP spots associated with topographically detectable buds between 18 and 24 h posttransfection. The remaining eight cells produced mostly uniform intracellular or indistinct fluorescence patches that did not match VLP-like topographical structures. The median height and diameter of the Gag-GFP–positive buds was 110 nm (IQR = 140 to 100, *n* = 39) and 140 nm (IQR = 160 to 125, n = 39), respectively, similar to VLP sizes formed on Jurkat cells transfected with pH2B-mPlum/Gag (*SI Appendix*, Fig. S1*G*). In agreement with SICM-FCM observations, TEM images of cells positive for Gag-GFP fluorescence indicated the presence of predominantly flat or curved Gag assemblies (Fig. 2*D*), although detailed examination of TEM images revealed discontinuities in the VLP Gag layer similar to previously published structures (18) and presumably caused by the presence of GFP (Fig. 2D, *Right*, arrow).

Given the low probability of detecting buds that have associated Gag-GFP fluorescence we increased the scan size of the SICM-FCM time-lapse images to 4 × 4 μm. This reduced the frame rate to one frame per minute. In 83 cells from 22 independent experiments we recorded a total of 25 events where a clear correlation between Gag-GFP fluorescence and topographically detected budding structures was observed from the nucleation of a bud to its disappearance along with its associated Gag-GFP fluorescence (Fig. 2*E*). The appearance of Gag-GFP fluorescence at a detectable level preceded the formation of buds, indicating progressive assembly of Gag into VLPs (Fig. 2*F*, *Inset*). We recorded an average budding event lifetime of 134 ± 30 s (Fig. 2*F*). Although this was limited by the frame rate, it is similar to that of the slower VLP population observed in cells transfected with H2B-mPlum/Gag.

**Fig. 3.**
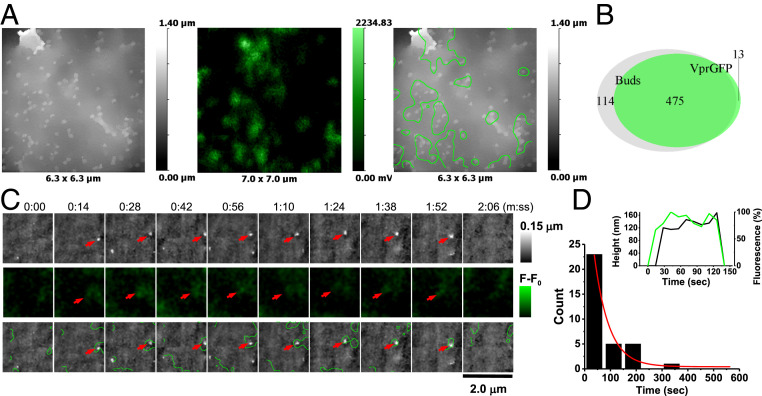
HIV VLP formation in Jurkat cells transfected with 1:1 Vpr-GFP and SynGP. (*A*) SICM-FCM images of a fixed cell showing correlation between Vpr-GFP fluorescence and VLP buds. (*B*) Euler diagram showing the relative overlap of Vpr-GFP fluorescence spots and topographically detected buds. (*C*) SICM-FCM time-lapse images showing the formation and disappearance of a VLP (red arrows). Images were acquired at 4.3 frames per minute. (*D*) Distribution of lifetimes of Vpr-GFP positive buds. (*Inset*) Bud height (black line) and fluorescence intensity (green line) traces for the VLP in *C*.

To attempt to reduce any potential interference of a fluorescent tag with VLP formation and improve the correlation between topographically detectable VLP buds and fluorescent signal, we tested whether Viral Protein R (Vpr) labeled with GFP (Vpr-GFP) could be used as an alternative marker of virion assembly. Vpr is not an HIV structural protein but is incorporated into virions at relatively low levels (compared to Gag) and has been used to carry fluorescent proteins to label infectious virus particles (19). According to the literature, the number of Vpr molecules per virion can range from 250 to 500 in transfected cells depending on Vpr expression level (20, 21). Although Vpr-GFP fluorescence levels were noticeably lower than those of Gag-GFP, which made correlation less reliable, 1:1 Vpr-GFP:SynGP resulted in efficient formation of topographically detectable buds that correlated with Vpr-GFP fluorescence spots. We detected correlation in 5 out of 13 cells fixed at 18 h and 7 out of 9 cells fixed at 24 h posttransfection (Fig. 3*A*). In these 12 cells we identified a total of 488 Vpr-GFP fluorescence spots and 489 topographically detected VLP buds, of which 475 overlapped. Therefore, over 97% of Vpr-GFP fluorescence spots correlated with budding structures and more than 80% of topographically detected buds had associated fluorescence (Fig. 3*B*). The median height and diameter of the buds was 110 nm (IQR = 130 to 90, *n* = 57) and 120 nm (IQR = 170 to 120, *n* = 57), respectively, similar to the sizes of VLPs formed in Jurkat cells transfected with pH2B-mPlum/Gag (*SI Appendix*, Fig. S1*G*).

In 75 cells from 18 independent experiments we identified and followed 34 budding structures that correlated with Vpr-GFP fluorescence from the moment of nucleation to disappearance at frame rates varying from 4.3 to 2 frames per minute (Fig. 3*C*). The distribution of VLP observation times best fitted with exponential decay and had a half-life of 40 s (Fig. 3*D*). This distribution is likely to be limited by the scan rate and the low probability of detecting successful budding events due to VLPs drifting in and out of the area of observation. For comparison, VLPs formed by unlabeled Gag shown in Fig. 1*H* had a half-life of 51 s when fitted exponentially. Similar to Gag-GFP, the appearance of Vpr-GFP fluorescence at a detectable level preceded the formation of topographically detectable buds, indicating early recruitment of Vpr into VLPs (Fig. 3*D*, *Inset*).

Together, these data indicate that budding of unlabeled and fluorescently tagged viruses can be followed in living cells at a single-particle level by SICM-FCM. Importantly, the kinetics of the topological changes associated with Gag-driven bud formation suggest that HIV VLP assembly and release may be quicker than previously reported using TIRFM (1–3). We also demonstrate that fluorescent labeling of Gag, for example by insertion of GFP, can alter Gag organization at the PM and influence the kinetics of VLP formation.

### VLP Formation in HeLa Cells.

**Fig. 4.**
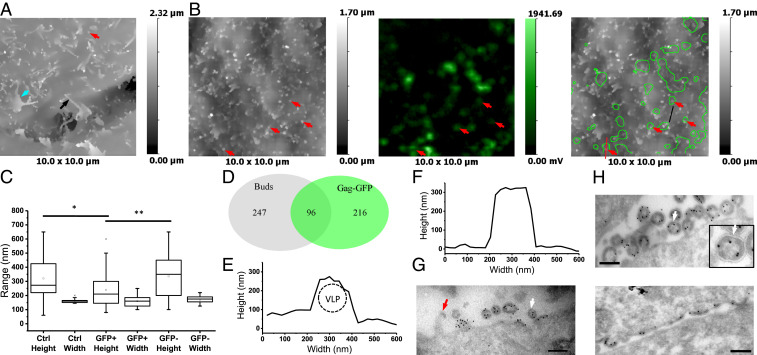
HIV VLP formation on HeLa cells. (*A*) SICM topographical image of an untransfected HeLa cell (fixed) showing membrane projections corresponding to short, stump-like microvilli (red arrow), long, finger-like microvilli (black arrow), and dorsal ruffles (cyan arrow). (*B*) Correlative SICM-FCM images of the top surface of a fixed HeLa cell transfected with Gag-GFP. Red arrows indicate membrane buds that have associated Gag-GFP fluorescence. (*C*) Stump-like projection and membrane bud heights and diameters (widths) measured in SICM topographical images of untransfected (*n* = 42, six cells) and Gag-GFP transfected cells, individually for buds with (*n* = 40) and without (*n* = 47) corresponding Gag-GFP fluorescence spots (five cells) (two-sample *t* test, *P < 0.05, **P < 0.01). (*D*) Euler diagram showing the relative overlap of Gag-GFP fluorescent spots and topographically detected buds. (*E*) Cross-section profile of a membrane protrusion corresponding to a typical Gag VLP measured in *B* (*Right*, red line). (*F*) Cross-section profile of a membrane protrusion corresponding to a typical microvillus in *D*, black line. (*G*) TEM image of immunolabeled cryosection of HeLa cells transfected with Gag-GFP. Red arrow indicates Gag at the tip of a microvillus. (*H*) TEM images of VLPs and Gag buds formed on unrestricted membrane (*Top*) and a restricted membrane in a cell–cell contact (*Bottom*), of the same HeLa cell transfected with Gag-SNAP. White arrows point at discontinuities in the Gag layer. Rabbit anti-p24/Protein A-gold is used in TEM images. (Scale bars: 200 nm.)

To compare our results with previously published observations, we analyzed VLP formation by SICM-FCM and TEM in HeLa cells, since the majority of TIRFM studies of HIV-1 assembly kinetics were undertaken in this cell type (1–3). However, topographical SICM images of the top surface of nontransfected, fixed HeLa cells revealed numerous membrane projections corresponding to microvilli and dorsal ruffles, some of which could be confused with fully formed buds and VLPs (Fig. 4*A*). We broadly classified these projections into three categories: 1) round, stump-like protrusions (red arrow) with median heights of 272.5 nm (median, IQR = 220 to 425, *n* = 42) and diameters of 160 nm (IQR = 150 to 165, *n* = 42) corresponding to microvilli, 2) finger-like protrusions (black arrow) corresponding to dorsal filopodia or microvilli, and 3) membrane flaps corresponding to ruffles (cyan arrow). The proportion of stump, finger-like microvilli, and dorsal ruffles varied significantly from cell to cell (*SI Appendix*, Fig. S2 *A* and *C*).

Correlative SICM-FCM imaging of HeLa cells transiently transfected with Gag-GFP also revealed numerous membrane protrusions (Fig. 4*B*, *Left*) and distinct fluorescence spots of Gag-GFP in surface confocal images (Fig. 4*B*, *Middle*). Superimposed fluorescence and topographical images indicated that many stump-like projections had associated Gag-GFP fluorescence (Fig. 4*B*, *Right*, red arrows). However, we also observed Gag-GFP fluorescence associated with finger-like microvilli and dorsal ruffles, as well as flat membrane areas (*SI Appendix*, Fig. S2 *B* and *D*). To find out whether the fluorescent stump-like projections could be VLPs we compared their heights and diameters with those of similar nonfluorescent projections in Gag-GFP–expressing cells and projections in nontransfected cells (Fig. 4*C*). We found that although the height of Gag-GFP–positive membrane protrusions was 210 nm (IQR = 145 to 300, *n* = 40), that is, nearly twice the diameter of an average VLP, it was also significantly lower compared to the heights of Gag-GFP–negative protrusions (350 nm, IQR = 200 to 450, *n* = 47). The high variation in the measured heights suggested that these structures were microvilli going through active growth or retraction at the moment of fixation (22). The diameters of Gag-GFP–positive and –negative protrusions were not significantly different (160 nm [IQR = 125 to 185, *n* = 40] and 175 nm [IQR = 155 to 190, *n* = 47], respectively). We identified a total of 312 Gag-GFP fluorescence spots and 343 topographically detected VLP buds, of which 96 overlapped (eight cells). Thus, 30% of the Gag-GFP fluorescence spots correlated with budding structures and 28% of the topographically detected buds had associated fluorescence (Fig. 4*D*). We conclude that the presence of Gag-GFP fluorescence is essential to reliably distinguish genuine VLPs from other topographically detected protrusions. For comparison, representative cross-section profiles for Gag-GFP positive and negative protrusions are shown in Fig. 4*E* and *F*, respectively.

TEM images of immunolabeled cryosections have shown that in HeLa cells Gag-GFP alone can produce VLPs with diameters up to 200 nm (Fig. 4*G*). This observation is in agreement with our SICM measurements, and with Müller et al. (6), arguing that it is not necessary to coexpress Gag and Gag-GFP to form VLPs in these cells. Also in agreement with previously reported observations (23), we occasionally observed Gag buds decorating the tips of microvilli (Fig. 4*G*, red arrow). This may explain the association of Gag-GFP fluorescence with finger-like protrusions in correlative SICM-FCM images.

As an alternative to GFP, we also analyzed SNAP-tagged Gag. SNAP is smaller than GFP (19.4 kDa vs. 26.9 kDa) and in this construct is inserted between the MA and CA domains of Gag and not at the C terminus. According to published data, this eases some of the steric constraints that impact on the assembly of Gag-GFP and produces particles similar to those produced by Gag alone (6). In HeLa cells transfected with Gag-SNAP, we observed budding of VLPs from both the unrestricted top surface of cells as well as surfaces closely opposed to adjacent cells. VLPs and buds formed on the unrestricted surface appeared as spherical/curved structures similar to those described above (Fig. 4*H*, *Top*), although some particles also contained interruptions in the Gag layer (Fig. 4*H*, *Inset*, white arrow). By comparison, budding structures on membranes of the same cell closely opposed to an adjoining cell appeared flat (Fig. 4*H*, *Bottom*). This could be due to a difference in the biophysical properties of the PM at cell–cell contacts and/or lack of an external space into which the VLP can bud. Regardless of the underlying reason, this suggests that the kinetics of VLPs assembly and budding at the basal surface, as measured by TIRFM, may be influenced by the close proximity of the glass substrate.

We attempted to follow the formation of VLPs in live Gag-GFP transfected HeLa cells. However, we found it difficult to reliably identify and follow individual VLPs due to the presence of dense and dynamic microvilli and dorsal ruffles.

### VLP Formation in Cos-7 Cells.

Unlike HeLa cells, Cos-7 cells develop very few microvilli and dorsal ruffles (24, 25), making these cells more reliable for the identification of other nanostructures, such as VLPs, in topographical studies (12). SICM images of Cos-7 cells fixed 26 h after transfection with H2B-mPlum/Gag (*SI Appendix*, Fig. S3*A*) revealed numerous membrane protrusions that resembled fully formed buds or VLPs with median height of 100 nm (IQR = 110 to 90, *n* = 31) and width of 100 nm (IQR = 110 to 90, *n* = 31). Both the height and width of the buds formed in Cos-7 cells were significantly lower (*P* < 0.05) than those of VLPs formed by unlabeled Gag in Jurkat cells. The density of protrusions indicated that, similar to Jurkat cells, many VLPs remained adhered to the cell membrane after the completion of assembly. TEM images of Cos-7 cells transfected with SynGP also confirmed the formation of similar-looking VLPs in this cell type (*SI Appendix*, Fig. S3 *B*, *Top*). Like in HeLa cells, Gag buds formed in cell–cell contact regions of Cos-7 cells were flat or lightly curved (*SI Appendix*, Fig. S3 *B*, *Bottom*). SICM time-lapse images recorded in Cos-7 cells 26 h posttransfection with H2B-mPlum/Gag showed that, similar to Jurkat cells, VLPs can assemble and reach full size in ∼30 s (*SI Appendix*, Fig. S3*C*, red arrows). Although Cos-7 cells express only low levels of tetherin (26) the majority of VLPs remained on the cell surface for tens of minutes, gradually drifting outside the observation area, preventing measurement of the time to release. It was not possible to image the protease-induced release of fully formed VLPs, or VLP formation in the presence of protease, because the protease treatment caused rapid changes in cell morphology followed by loss of adhesion and cell rounding.

SICM-FCM images of cells fixed 24 h posttransfection with 1:1 Gag-GFP:SynGP revealed both Gag-GFP–positive buds and protrusions without detectable Gag-GFP fluorescence (*SI Appendix*, Fig. S3*D*, red and white arrows, respectively). We identified a total of 363 Gag-GFP fluorescence spots and 123 topographically detected VLP buds of which 90 overlapped (10 cells), that is, 25% of the Gag-GFP fluorescence spots correlated with budding structures and over 70% of the topographically detected buds had associated fluorescence (*SI Appendix*, Fig. S3*E*). The median height of the Gag-GFP–negative protrusions was 160 nm (IQR = 200 to 80, *n* = 19), significantly higher (*P* < 0.001) than the Gag-GFP–positive buds (90 nm, IQR = 110 to 45, *n* = 31), suggesting that the former could be microvilli (*SI Appendix*, Fig. S4*C*). The median diameters (115 nm, IQR = 150 to 90, *n* = 31) of Gag-GFP–positive protrusions were significantly larger than the diameters of buds formed by unlabeled Gag (*P* < 0.008), suggesting that, in Cos-7 cells, GFP tagging interferes with Gag-driven VLP formation. Confirming this, TEM images of immunolabeled cryosections of Cos-7 cells with 1:1 Gag-GFP:SynGP revealed the formation of flat and lightly curved Gag assemblies (*SI Appendix*, Fig. S3 *F*, *Top*) or aberrantly shaped VLPs with discontinuities in the Gag layer (*SI Appendix*, Fig. S3 *F*, *Bottom*, arrow). To determine whether the flat Gag structures observed in SICM-FCM and TEM images of fixed cells represent an early stage of budding, and to measure the assembly and release time, we performed time-lapse SICM-FCM imaging in live Cos-7 cells transfected with 1:1 Gag-GFP:SynGP. In agreement with the observations in fixed cells, live SICM-FCM images revealed correlations between Gag-GFP fluorescence spots and putative flat or lightly curved Gag assemblies (*SI Appendix*, Fig. S3*G*) and fully developed VLP buds (*SI Appendix*, Fig. S3*A*). Gag-GFP fluorescence spots that correlated with flat or lightly curved Gag assemblies had a variety of lifetimes ranging from 20 s (*SI Appendix*, Fig. S3*G*, white arrow) to more than 15 min (*SI Appendix*, Fig. S3*G*, red arrow). Importantly, we did not observe progressive growth of flat or lightly curved Gag assemblies into fully formed buds (*n* = 84). The full-size Gag-GFP–positive protrusions were also static (*SI Appendix*, Fig. S4*A*), outlasting the duration of observation, and had an average lifetime of 1,266 ± 571 s (*n* = 7). For comparison, cross-section profiles of lightly curved and fully developed VLPs are presented in *SI Appendix*, Fig. S4*B*. The long lifetime of full-size buds suggests they represent either buds aborted at a late stage of assembly or fully assembled and released VLPs that remain attached to the PM. Transfection of Cos-7 cells with a 1:5 ratio of Gag-GFP and SynGP as well as with 1:1 Vpr-GFP:SynGP also resulted in the formation of mainly flat and lightly curved Gag assemblies (*SI Appendix*, Fig. S4 *D*–*G*).

These data indicate that VLP formation by unlabeled Gag may take considerably less time than previously estimated and that fluorescent tagging may affect VLP structure formation. Also, compared to Jurkat cells, in Cos-7 cells the majority of buds remained shallow and fully formed VLPs were rarely observed.

### VLP Formation in HEK293T Cells.

**Fig. 5.**
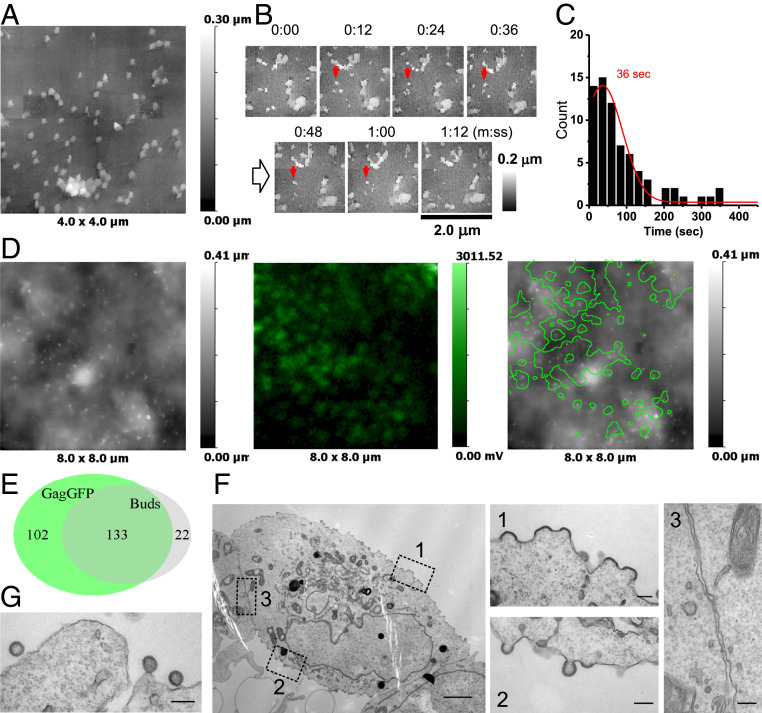
HIV VLPs on HEK293T cells. (*A*) SICM image of VLPs on a cell transfected with H2B-mPlum/Gag fixed at 20 h posttransfection. (*B*) HS-SICM time-lapse images showing the formation and disappearance of VLPs (red arrows) in cells recorded starting from 8 h posttransfection. Images were acquired at five frames per minute. (*C*) Distribution of VLP lifetimes. (*D*) Correlative SICM-FCM images of budding structures on fixed cell transfected with 1:5 Gag-GFP:SynGP. (*E*) Euler diagram showing the distribution overlap of Gag-GFP fluorescence spots and topographically detected buds. (*F*) TEM images of transfected HEK293T cells; high-magnification views show buds formed on unrestricted membranes (1 and 2) and no buds formed on membranes closely opposed to adjacent cells (3). (*G*) TEM image of VLPs on cell transfected with SynGP only. (Scale bars: 2 μm in *F*, *Left* and 200 nm in *F* [high-magnification panels] and *G*.)

HEK293T cells are frequently used to produce recombinant infectious lentiviruses, and we therefore also examined the formation of Gag VLPs in these cells. SICM topographical images of cells fixed at 20 h posttransfection with H2B-mPlum/Gag revealed PM protrusions that by size and shape resembled either fully formed buds or cell-surface-adhered VLPs (Fig. 5*A*). The protrusions had median height of 104 nm (IQR = 110 to 100, *n* = 31) and width of 103 nm (IQR = 115 to 90, *n* = 31) (*SI Appendix*, Fig. S5), similar to the dimensions of buds formed by unlabeled Gag in Cos-7 cells and significantly lower (*P* < 0.05) than in Jurkat cells. Protease treatment of cells with 100 μg/mL *B. licheniformis* Subtilisin A resulted in a rapid 64 ± 13% (two independent experiments) reduction in the number of protrusions, indicating that they represented VLPs that remained adhered to the cell surface after budding is complete. Similar to Cos-7, tetherin expression is reported to be undetectable in HEK293T cells (27). SICM time-lapse images of cells recorded 25 to 27 h after transfection, in the presence of protease (one cell, one experiment), revealed actively forming VLPs (Fig. 5*B*). The average lifetime of buds was 36 ± 43 s (Fig. 5*C*), similar to that of buds formed by unlabeled Gag in Jurkat cells.

SICM-FCM imaging of HEK293T cells transfected with 1:5 Gag-GFP:SynGP revealed that the majority of membrane protrusions with associated GFP fluorescence were lightly curved or hemispherical in shape (Fig. 5*D*) with a median height of 54 nm (IQR = 77.5 to 27.5, *n* = 52, 5 cells) and a diameter of 145 nm (IQR = 150 to 112.5, *n* = 52, 5 cells) (*SI Appendix*, Fig. S5). We identified a total of 235 Gag-GFP fluorescence spots and 155 topographically detected VLP buds, of which 133 overlapped (10 cells), that is, 56% of all Gag-GFP fluorescence spots correlated with topographically detectable membrane protrusions and only 14% of buds were GFP-negative (Fig. 5*E*). In accordance with SICM-FCM, TEM images indicated numerous hemispherical and occasional lollypop-shaped Gag buds on unrestricted membranes (Fig. 5*F*, high-magnification panels 1 and 2). However, fully formed PM-associated VLPs were rare. We did not observe budding structures on membranes closely opposed to adjacent cells (Fig. 5*F*, panel 3). Similar to Cos-7 cells, transfection of HEK293T cells with SynGP confirmed that VLPs with shapes and sizes similar to wild-type viruses were formed and released (Fig. 5*G*).

**Fig. 6.**
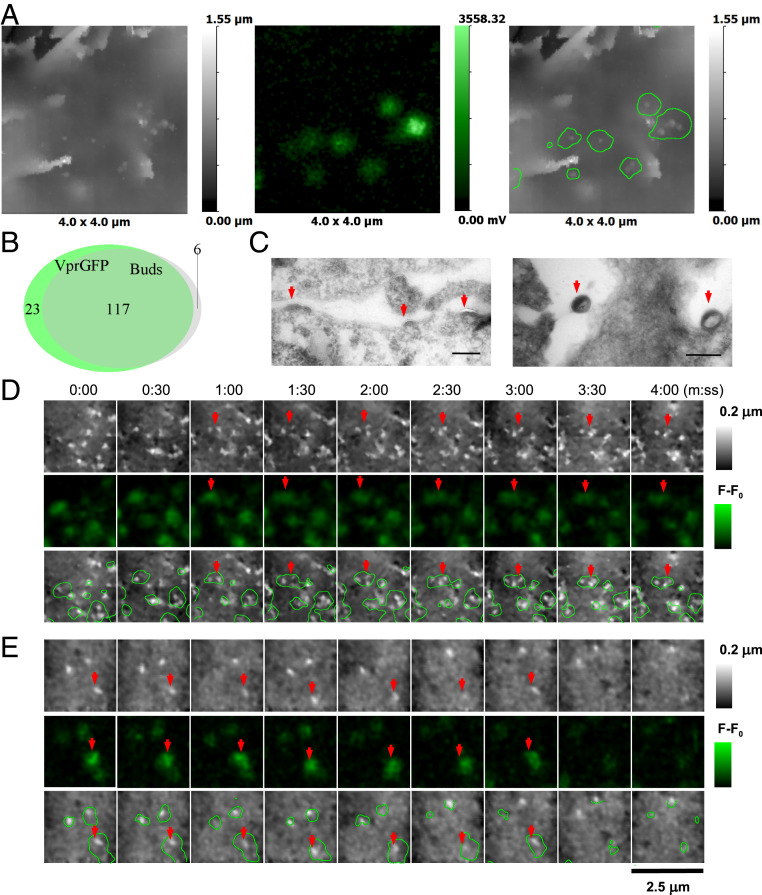
HIV VLP formation in HEK293T cells transfected with Vpr-GFP and SynGP. (*A*) Correlative SICM-FCM images of VLPs on fixed HEK293T cells transfected with 1:1 Vpr-GFP:SynGP. (*B*) Euler diagram showing the relative overlap of Vpr-GFP fluorescence spots with topographically detected buds. (*C*) TEM images of a cell transfected with 1:1 Vpr-GFP:SynGP showing flat Gag assemblies and released VLPs (red arrows). (Scale bars: 200 nm.) (*D* and *E*) SICM-FCM time-lapse images showing the formation (*D*, red arrows) and disappearance of VLP (*E*, red arrows). Images acquired at 2 frames per minute.

Transfection of HEK293T cells with 1:1 Vpr-GFP:SynGP resulted in the formation of buds (Fig. 6*A*) with the mean height of 140 nm (IQR = 160 to 120, *n* = 43, six cells) and diameter of 105 nm (IQR = 120 to 100, *n* = 43, six cells) (*SI Appendix*, Fig. S5). We identified a total of 140 Gag-GFP fluorescence spots and 123 topographically detected VLP buds, of which 117 overlapped (nine cells), that is, 84% of all Vpr-GFP fluorescence spots correlated with topographically detectable membrane protrusions and 95% of buds were Gag-GFP–positive (Fig. 6*B*). TEM images showed occasional PM Gag assemblies ranging from flat to fully formed VLPs (Fig. 6*C*). In SICM-FCM time-lapse images we also observed significant correlation between topographically detectable buds and Vpr-GFP fluorescence (Fig. 6*D* and *E*), although it was not always possible to resolve individual fluorescence spots as buds were often spaced closer than the optical diffraction limit. Although we were able to detect the appearance of newly forming buds (Fig. 6*D*, red arrows) as well as VLP disappearance (Fig. 6*E*, red arrows), it was not possible to follow the entire tracks as the majority of buds remained on the cell surface for longer than 30 min (*n* = 22) and drifted in and out of the scan area. Some buds were observed on the PM for as long as 2 h. The long residence times of full-size buds either indicates that they were arrested at a late stage of budding or remained bound to the cell surface after budding was completed.

The data obtained in HEK293T cells suggest that fluorescent tagging may affect VLP structure and assembly kinetics that echoes the results obtained in Cos-7 and Jurkat cells.

### Cell Membrane Tension and Viscosity in VLP Formation.

Cell membranes are best described as a two-dimensional viscoelastic material/non-Newtonian fluid that exhibits both viscous and elastic properties when deformed (28, 29). In an attempt to understand how the biophysical properties of the PM affect VLP formation, we mapped cell stiffness using SICM and measured membrane microviscosity by fluorescence lifetime imaging microscopy (FLIM) in all four cell types used in the study.

It has been previously demonstrated that a SICM pipette can be used to measure cell stiffness and droplet surface tension without coming into direct contact with the sample surface. This can be achieved by deforming the PM either by using hydrostatic pressure to fire a jet of liquid from the tip of the pipette (30, 31) or by using intrinsic repulsion forces that appear in close-range interactions between the negatively charged glass of the pipette and negatively charged lipid membrane. These interactions occur at high set points (e.g., 2 to 3%) compared to the set point typically used for imaging, that is, 0.4 to 0.5% of ion current drop (32, 33). In our experiments we used the second method as it allowed us to use smaller-diameter pipettes and thereby achieve higher spatial resolution when mapping cell stiffness. It has been shown that at 2% set point a 110-nm-aperture pipette exerts sufficient force to push the PM 100 to 200 nm, not enough to deform the underlying cytoskeleton. A 3% set point is necessary to create a higher force required to measure the stiffness of the underlying actin cortex (32). Thus, low-force stiffness mapping can be used to report PM tension. We mapped the stiffness in control cells and cells transfected with H2B-mPlum/Gag, both positive and negative for H2B-mPlum fluorescence. Typical cell topography images and corresponding stiffness maps of cells positive for H2B-mPlum fluorescence are shown in *SI Appendix*, Fig. S6 *A*–*D*. Statistical analysis revealed that the stiffness of control Jurkat cells was significantly lower than the stiffness of other three cell types, and the stiffness of HEK293T cells was lower than the stiffness of Cos-7 and HeLa cells (two-sample *t* test, *P* < 0.01). Of all of the cell lines Gag expression significantly reduced the cell stiffness only in HeLa cells (two-sample *t* test, *P* < 0.04) (*SI Appendix*, Fig. S6*E*). Thus, the probability of detecting fluorescently labeled VLP formation correlated with lower PM tension.

We then imaged PM viscosity by FLIM using membrane-inserting BODIPY-based molecular rotors as previously described (34, 35). It has been demonstrated that BODIPY rotors localize in the acyl chain regions of fluid-phase bilayers (36) and, upon excitation, can either undergo radiative decay via fluorescence emission or decay via nonradiative pathways, typically involving an intramolecular rotation mechanism. The rate of this nonradiative decay is influenced by the friction imposed by the surrounding environment, that is, the local microviscosity (37, 38). We acquired FLIM images sampling separately top, nontouching membranes and middle (equatorial) membranes where cells form contacts. It was not possible to reliably measure BODIPY lifetime at the bottom membranes, near the surface of the coverslip, due to laser scattering at the glass/liquid interface. Representative FLIM images of the top and middle membranes of Jurkat and HEK293T cells transfected with H2B-mPlum/Gag are shown in *SI Appendix*, Fig. S7 *A* and *C*, *Left* and *Right*, respectively. We found that the viscosity of the top, nontouching membranes was similar in all cell types (*SI Appendix*, Fig. S7 *F*, *Left*). Intriguingly, similar to the reduction in cell stiffness, Gag expression only influenced top membrane viscosity in HeLa cells. The viscosity of membranes at cell–cell contacts was also similar in all cell types (*SI Appendix*, Fig. S7 *F*, *Right*) and was unaffected by Gag expression. However, with the exception of HeLa cells, the viscosity of the PM at cell–cell contacts was significantly higher than the viscosity of the top, nontouching membranes (two-sample *t* test, *P* < 0.01). An example of higher membrane viscosity at cell–cell contact in Jurkat cells is shown in *SI Appendix*, Fig. S7 *A*, *Left*, arrow. This correlates well with our observation that only flat or lightly curved Gag structures were observed by TEM at cell–cell contacts.

## Discussion

For many enveloped viruses, particle assembly occurs at the PM, so that on completion free virions are released to the extracellular space. Although recognized as an essential step in virus replication and transmission, the dynamics of particle formation for most viruses are poorly understood. For HIV-1, assembly and budding have been studied in detail using fluorescence live-cell imaging techniques (1–3, 5) which, for the most part, are based on TIRF imaging where the observed particles are formed on a cell membrane adjacent to a glass substrate. Although this approach allows better visualization of assembly, by reducing background fluorescence, contact with glass surfaces has been reported to influence the properties of the PM, inducing receptor clustering, mobility changes (39) and cytoskeletal rearrangements (40) that may influence the dynamics of virus assembly and release. Thus, alternative methods that can image virus formation on nonadherent cell surfaces may provide additional information on virus assembly and release. Here we show that HS correlative SICM-FCM can be used to visualize both unlabeled and fluorescently labeled VLP formation at the top, nonadherent surface of transfected cells. Importantly, this technique can monitor not only the recruitment of fluorescently tagged viral proteins to budding sites but also measure the topological changes associated with membrane deformation and particle release.

We found that on the nonadhered membrane of Jurkat, Cos-7, and HEK293T cells, the topological changes associated with VLP formation can occur in as little as 20 s. The majority of fully assembled VLPs in Jurkat and HEK293T cells are released in 0.5 to 3 min following the first detection of these topological changes. Although it has recently been demonstrated that depletion of PIP_2_ results in disassembly of Gag lattices from the cell membrane (16), we assumed that Gag assembly into buds under physiological conditions is irreversible and the abrupt disappearance of topographically detected buds together with associated fluorescence represents VLP departure. This is significantly faster than reported previously (1, 3, 5). Although Gag-GFP fluorescence puncta with lifetimes of 33 s were previously reported, on the basis of colocalization with endocytic markers, they were not considered budding VLPs (3). Contrary to our expectations, we did not observe a gradual bud height increase over a course of ∼8 min that would be indicative of progressive Gag assembly into VLPs followed by particle release at ∼25 min after the onset of assembly in accordance with previously published observations (1–3, 5, 41, 42). To compare our measurements with published data we calculated VLP budding times for complete events from the moment a fluorescence spot first appeared to the point of disappearance of topographically detected VLPs. The average budding time was 248 ± 155 s (*n* = 11) in Jurkat cells transfected with Gag-GFP:SynGP 1:1 and 113 ± 81 s (*n* = 12) in cells transfected with Vpr-GFP:SynGP 1:1, which is significantly faster than published previously. For events in which the release of a VLP was not registered, due to VLPs drifting out of the area of observation, we calculated the time delay between the first appearance of fluorescence signal and the first appearance of a topologically detectable bulge. In 12 out of 19 events identified for both constructs the fluorescence signals appeared simultaneously with the buds. The average time delay was 45 ± 99 s (*n* = 8) in Jurkat cells transfected with Gag-GFP:SynGP 1:1 and 33 ± 60 s (*n* = 11) in cells transfected with Vpr-GFP:SynGP 1:1. For unlabeled VLPs we estimated the time delay as follows. An average VLP with a radius *r* = 60 nm, and therefore a surface area equal to 45,239 nm^2^, is typically made of 2,000 to 5,000 Gag molecules, that is, 9 to 22.6 nm^2^ per Gag molecule. The surface area of a minimal bud (spherical cap, height = 10 nm) would be 3,777 nm^2^ and contain 167 to 419 Gag molecules. Considering the assembly rate of 5 to 10 Gags per s (43) it would take 17 to 84 s to form a 10-nm-height bud that could be resolved by SICM with confidence. This time is very close to the time delay between the appearance of fluorescence signal and the first appearance of a bulge that we measured experimentally for labeled VLPs. When these time delays are added to the average budding times of fluorescently tagged and unlabeled VLPs, calculated from SICM topographical data, the budding times are still significantly faster than previously published. It is possible that in our experiments we overlooked a substantial fraction of particles with longer lifetimes as they drifted out of the observation area. Increasing the scan area was found impractical as it results in a reduction of the scan rate (a drawback that is true for all scanning probe microscopy techniques), preventing reliable tracking of VLPs.

The much faster assembly kinetics we observed could possibly be explained by the fact that we followed VLP formation on the top surfaces of cells where assembly is not restricted by the close proximity of a glass substrate. Although it has been reported that the average time from initiation of assembly to release of HIV-1 from the dorsal surface of cells, recorded by spinning-disk confocal microscopy, was indistinguishable from the results obtained with TIRFM (1), our TEM data showing flat Gag patches in space-restricted areas support the idea that free space may influence the kinetics of virus assembly. Another possibility is that the longer assembly and release times observed by fluorescence imaging were due to nonspecific binding of newly formed VLPs to cell membranes (1, 5). This is supported by our observation that on protease treatment of VLP producing Jurkat and HEK293T cells, that do not express tetherin or TIM-1, at least three-quarters of VLPs may remain surface-bound after assembly was completed.

We also found that VLP formation can be influenced by fluorescence labeling techniques and the efficiency of fluorescently labeled particle formation may vary in different cell types. It is difficult to estimate to what degree GFP affects the VLP budding and release kinetics as the exact proportion of Gag-GFP molecules in each bud varies. The cotransfection approach used to avoid morphological defects observed in VLPs when using Gag-GFP alone was recently scrutinized by Gunzenhäuser et al. (7). The authors argued that, for a given cotransfection ratio (when using more than one construct), individual cells express a wide range of protein ratios and that quantification of unlabeled Gag expression is required. Moreover, a clear correlation between the amount of unlabeled Gag within a single cell and the number of labeled Gag proteins per cluster has not been reported. In line with this, our results show that although Gag-GFP clusters in cell membranes produce diffraction-limited fluorescence spots with a breadth of lifetimes, this does not guarantee the subsequent formation of topographically detectable buds and VLPs. Although reduction of pGag_eGFP when cotransfected with pSynGP caused lower expression of the corresponding protein, as judged by GFP fluorescence, it did not rescue VLP budding in Cos-7 and HEK293T cells.

The observation of different VLP formation properties in different cell types led us to investigate the biophysical properties of the PMs. Our cell stiffness and membrane microviscosity data show that the assembly of topographically detectable fluorescently labeled VLPs is more efficient in softer cells (cells with lower membrane tension) such as Jurkat, and in areas with lower membrane viscosity and free outer space, that is, away from cell–cell contacts. According to theoretical models, Gag–Gag interaction results in a line tension at the rim of a partially formed bud that is sufficient to counteract the force caused by bending rigidity and tension of the lipid membrane, enabling HIV assembly to proceed to completion (44). However, the bending rigidity and viscosity depend on lipid composition of the membrane, which is influenced by, for example, acyl chain length, cholesterol content, and so on that varies in different cell types (45, 46). It has previously been demonstrated that bud growth time increases exponentially with increasing membrane viscosity (44). Therefore, assembly events can be trapped as partial buds by reducing Gag–Gag attraction or by increasing membrane tension. Similar to previous observations (18), our TEM data revealed GFP-associated discontinuities in the Gag layer of buds and VLPs in all cell types used in this study. Such discontinuities may reduce the forces generated by Gag–Gag interaction required to counteract membrane tension and hinder VLP assembly. In agreement with this, we found that compared to coexpression of Gag-GFP and SynGP where GFP may interfere with Gag–Gag interaction, the transfection with Vpr-GFP and SynGP consistently resulted in higher correlation between topographically detected buds and fluorescence in Jurkat and HEK293T cells.

In summary, we show that HS correlative SICM-FCM provides a methodology for live imaging the formation of virus particles budding from cell PMs. This approach can be applied to normally nonadherent cells, such as Jurkat T cells, a physiologically relevant model for HIV replication, and to a variety of adherent cell types. Using this approach, we find that the assembly and release of HIV VLPs may take significantly less time than previously thought and may vary for different PM domains. The VLP assembly and release kinetics we observed are not dissimilar to those of clathrin-coated vesicles, which, judged purely by the total number of molecules involved, is somewhat more complicated than HIV formation (47, 48).

## Materials and Methods

Jurkat, HeLa, Cos7, or HEK293T cells were transfected using Lipofectamine (Invitrogen) according to the manufacturer’s protocol. Correlative SICM-FCM live imaging was performed in Hanks’ balanced salt solution (HBSS) (Gibco) supplemented with 10 mM Hepes (Sigma) using a custom-built SICM setup (ICAPPIC Ltd). For TEM imaging cells were fixed with 3% formaldehyde and 2% sucrose dissolved in HBSS, 10 mM Hepes, and 1% glutamax for 30 min and subsequently in 2% formaldehyde and 1.5% glutaraldehyde in HBSS for another 30 min. For detailed materials and methods see *SI Appendix*.

## Supplementary Material

Supplementary File

## Data Availability

All study data are included in the article and *SI Appendix*.

## References

[1] Ivanchenko S.., Dynamics of HIV-1 assembly and release. PLoS Pathog. 5, e1000652 (2009). 19893629 10.1371/journal.ppat.1000652 PMC2766258

[2] Ku P. I.., Identification of pauses during formation of HIV-1 virus like particles. Biophys. J. 105, 2262–2272 (2013). 24268138 10.1016/j.bpj.2013.09.047 PMC3838742

[3] Jouvenet N., Bieniasz P. D., Simon S. M., Imaging the biogenesis of individual HIV-1 virions in live cells. Nature 454, 236–240 (2008). 18500329 10.1038/nature06998 PMC2708942

[4] Gheysen D.., Assembly and release of HIV-1 precursor Pr55gag virus-like particles from recombinant baculovirus-infected insect cells. Cell 59, 103–112 (1989). 2676191 10.1016/0092-8674(89)90873-8

[5] Larson D. R., Johnson M. C., Webb W. W., Vogt V. M., Visualization of retrovirus budding with correlated light and electron microscopy. Proc. Natl. Acad. Sci. U.S.A. 102, 15453–15458 (2005). 16230638 10.1073/pnas.0504812102 PMC1266096

[6] Müller B.., Construction and characterization of a fluorescently labeled infectious human immunodeficiency virus type 1 derivative. J. Virol. 78, 10803–10813 (2004). 15367647 10.1128/JVI.78.19.10803-10813.2004 PMC516407

[7] Gunzenhäuser J., Wyss R., Manley S., A quantitative approach to evaluate the impact of fluorescent labeling on membrane-bound HIV-Gag assembly by titration of unlabeled proteins. PLoS One 9, e115095 (2014). 25493438 10.1371/journal.pone.0115095 PMC4262470

[8] Novak P.., Nanoscale live-cell imaging using hopping probe ion conductance microscopy. Nat. Methods 6, 279–281 (2009). 19252505 10.1038/nmeth.1306 PMC2702483

[9] Shevchuk A. I.., Imaging proteins in membranes of living cells by high-resolution scanning ion conductance microscopy. Angew. Chem. Int. Ed. Engl. 45, 2212–2216 (2006). 16506257 10.1002/anie.200503915

[10] Novak P.., Imaging single nanoparticle interactions with human lung cells using fast ion conductance microscopy. Nano Lett. 14, 1202–1207 (2014). 24555574 10.1021/nl404068p

[11] Shevchuk A. I.., An alternative mechanism of clathrin-coated pit closure revealed by ion conductance microscopy. J. Cell Biol. 197, 499–508 (2012). 22564416 10.1083/jcb.201109130 PMC3352948

[12] Shevchuk A. I.., Imaging single virus particles on the surface of cell membranes by high-resolution scanning surface confocal microscopy. Biophys. J. 94, 4089–4094 (2008). 18199668 10.1529/biophysj.107.112524 PMC2367192

[13] Freed E. O., Delwart E. L., Buchschacher G. L. Jr., Panganiban A. T., A mutation in the human immunodeficiency virus type 1 transmembrane glycoprotein gp41 dominantly interferes with fusion and infectivity. Proc. Natl. Acad. Sci. U.S.A. 89, 70–74 (1992). 1729720 10.1073/pnas.89.1.70 PMC48177

[14] Perez-Caballero D.., Tetherin inhibits HIV-1 release by directly tethering virions to cells. Cell 139, 499–511 (2009). 19879838 10.1016/j.cell.2009.08.039 PMC2844890

[15] Weinelt J., Neil S. J. D., Differential sensitivities of tetherin isoforms to counteraction by primate lentiviruses. J. Virol. 88, 5845–5858 (2014). 24623426 10.1128/JVI.03818-13 PMC4019096

[16] Binné L. L., Scott M. L., Rennert P. D., Human TIM-1 associates with the TCR complex and up-regulates T cell activation signals. J. Immunol. 178, 4342–4350 (2007). 17371991 10.4049/jimmunol.178.7.4342

[17] Kotsopoulou E., Kim V. N., Kingsman A. J., Kingsman S. M., Mitrophanous K. A., A Rev-independent human immunodeficiency virus type 1 (HIV-1)-based vector that exploits a codon-optimized HIV-1 gag-pol gene. J. Virol. 74, 4839–4852 (2000). 10775623 10.1128/jvi.74.10.4839-4852.2000 PMC112007

[18] Pornillos O.., HIV Gag mimics the Tsg101-recruiting activity of the human Hrs protein. J. Cell Biol. 162, 425–434 (2003). 12900394 10.1083/jcb.200302138 PMC2172688

[19] McDonald D.., Visualization of the intracellular behavior of HIV in living cells. J. Cell Biol. 159, 441–452 (2002). 12417576 10.1083/jcb.200203150 PMC2173076

[20] Singh S. P.., Virion-associated HIV-1 Vpr: Variable amount in virus particles derived from cells upon virus infection or proviral DNA transfection. Virology 283, 78–83 (2001). 11312664 10.1006/viro.2001.0849

[21] Lai D.., Extent of incorporation of HIV-1 Vpr into the virus particles is flexible and can be modulated by expression level in cells. FEBS Lett. 469, 191–195 (2000). 10713269 10.1016/s0014-5793(00)01264-3

[22] Gorelik J.., Dynamic assembly of surface structures in living cells. Proc. Natl. Acad. Sci. U.S.A. 100, 5819–5822 (2003). 12721367 10.1073/pnas.1030502100 PMC156284

[23] Stauffer S.., The nucleocapsid domain of Gag is dispensable for actin incorporation into HIV-1 and for association of viral budding sites with cortical F-actin. J. Virol. 88, 7893–7903 (2014). 24789788 10.1128/JVI.00428-14 PMC4097806

[24] Porter K. R., Fonte V., Weiss G., A scanning microscope study of the topography of HeLa cells. Cancer Res. 34, 1385–1394 (1974). 4857013

[25] Yonemura S., Matsui T., Tsukita S., Tsukita S., Rho-dependent and -independent activation mechanisms of ezrin/radixin/moesin proteins: An essential role for polyphosphoinositides in vivo. J. Cell Sci. 115, 2569–2580 (2002). 12045227 10.1242/jcs.115.12.2569

[26] Waheed A. A., Kuruppu N. D., Felton K. L., D’Souza D., Freed E. O., In COS cells Vpu can both stabilize tetherin expression and counteract its antiviral activity. PLoS One 9, e111628 (2014). 25360760 10.1371/journal.pone.0111628 PMC4216104

[27] Van Damme N.., The interferon-induced protein BST-2 restricts HIV-1 release and is downregulated from the cell surface by the viral Vpu protein. Cell Host Microbe 3, 245–252 (2008). 18342597 10.1016/j.chom.2008.03.001 PMC2474773

[28] Harland C. W., Bradley M. J., Parthasarathy R., Phospholipid bilayers are viscoelastic. Proc. Natl. Acad. Sci. U.S.A. 107, 19146–19150 (2010). 20974934 10.1073/pnas.1010700107 PMC2984223

[29] Evans E. A., Hochmuth R. M., Membrane viscoelasticity. Biophys. J. 16, 1–11 (1976). 1244886 10.1016/S0006-3495(76)85658-5 PMC1334809

[30] Rheinlaender J., Schäffer T. E., Mapping the mechanical stiffness of live cells with the scanning ion conductance microscope. Soft Matter 9, 3230–3236 (2013).

[31] Sánchez D.., Noncontact measurement of the local mechanical properties of living cells using pressure applied via a pipette. Biophys. J. 95, 3017–3027 (2008). 18515369 10.1529/biophysj.108.129551 PMC2527257

[32] Clarke R. W.., Low stress ion conductance microscopy of sub-cellular stiffness. Soft Matter 12, 7953–7958 (2016). 27604678 10.1039/c6sm01106c PMC5166566

[33] Clarke R. W.., Pipette-surface interaction: Current enhancement and intrinsic force. J. Am. Chem. Soc. 135, 322–329 (2013). 23210472 10.1021/ja3094586

[34] Kubánková M., Summers P. A., López-Duarte I., Kiryushko D., Kuimova M. K., Microscopic viscosity of neuronal plasma membranes measured using fluorescent molecular rotors: Effects of oxidative stress and neuroprotection. ACS Appl. Mater. Interfaces 11, 36307–36315 (2019). 31513373 10.1021/acsami.9b10426

[35] Kubánková M., López-Duarte I., Kiryushko D., Kuimova M. K., Molecular rotors report on changes in live cell plasma membrane microviscosity upon interaction with beta-amyloid aggregates. Soft Matter 14, 9466–9474 (2018). 30427370 10.1039/c8sm01633j

[36] Dent M. R.., Imaging phase separation in model lipid membranes through the use of BODIPY based molecular rotors. Phys. Chem. Chem. Phys. 17, 18393–18402 (2015). 26104504 10.1039/c5cp01937k

[37] Vyšniauskas A., Kuimova M. K., A twisted tale: Measuring viscosity and temperature of microenvironments using molecular rotors. Int. Rev. Phys. Chem. 37, 259–285 (2018).

[38] Kuimova M. K., Mapping viscosity in cells using molecular rotors. Phys. Chem. Chem. Phys. 14, 12671–12686 (2012). 22806312 10.1039/c2cp41674c

[39] James J. R.., The T cell receptor triggering apparatus is composed of monovalent or monomeric proteins. J. Biol. Chem. 286, 31993–32001 (2011). 21757710 10.1074/jbc.M111.219212 PMC3173209

[40] Haghparast S. M. A., Kihara T., Miyake J., Distinct mechanical behavior of HEK293 cells in adherent and suspended states. PeerJ. 3, e1131 (2015). 26246972 10.7717/peerj.1131 PMC4525692

[41] Jouvenet N., Simon S. M., Bieniasz P. D., Imaging the interaction of HIV-1 genomes and Gag during assembly of individual viral particles. Proc. Natl. Acad. Sci. U.S.A. 106, 19114–19119 (2009). 19861549 10.1073/pnas.0907364106 PMC2776408

[42] Floderer C.., Single molecule localisation microscopy reveals how HIV-1 Gag proteins sense membrane virus assembly sites in living host CD4 T cells. Sci. Rep. 8, 16283 (2018). 30389967 10.1038/s41598-018-34536-y PMC6214999

[43] Tomasini M. D., Johnson D. S., Mincer J. S., Simon S. M., Modeling the dynamics and kinetics of HIV-1 Gag during viral assembly. PLoS One 13, e0196133 (2018). 29677208 10.1371/journal.pone.0196133 PMC5909904

[44] Zhang R., Nguyen T. T., Model of human immunodeficiency virus budding and self-assembly: Role of the cell membrane. Phys. Rev. E Stat. Nonlin. Soft Matter Phys. 78, 051903 (2008). 19113151 10.1103/PhysRevE.78.051903

[45] Rawicz W., Olbrich K. C., McIntosh T., Needham D., Evans E., Effect of chain length and unsaturation on elasticity of lipid bilayers. Biophys. J. 79, 328–339 (2000). 10866959 10.1016/S0006-3495(00)76295-3 PMC1300937

[46] Koster G., VanDuijn M., Hofs B., Dogterom M., Membrane tube formation from giant vesicles by dynamic association of motor proteins. Proc. Natl. Acad. Sci. U.S.A. 100, 15583–15588 (2003). 14663143 10.1073/pnas.2531786100 PMC307611

[47] Sundquist W. I., Kräusslich H.-G., HIV-1 assembly, budding, and maturation. Cold Spring Harb. Perspect. Med. 2, a006924 (2012). 22762019 10.1101/cshperspect.a006924 PMC3385941

[48] McMahon H. T., Boucrot E., Molecular mechanism and physiological functions of clathrin-mediated endocytosis. Nat. Rev. Mol. Cell Biol. 12, 517–533 (2011). 21779028 10.1038/nrm3151

