# Supplementary material for: Rapid formation of human immunodeficiency virus-like particles
Source: Proc Natl Acad Sci U S A. 2020 Aug 17;117(35):21637–46. doi: 10.1073/pnas.2008156117 (PMC7474690; doi:10.1073/pnas.2008156117)
Supplement: Supplementary File [file pnas.2008156117.sapp.pdf]

Supplementary Information for

Rapid formation of human immunodeficiency virus like particles

Bednarska et. al.

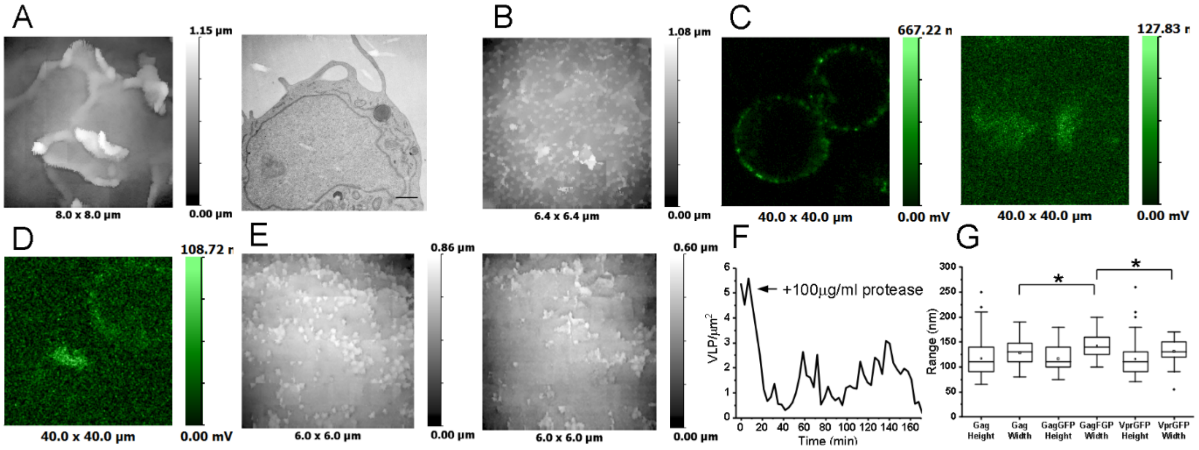

Figure S1. VLP formation in Jurkat cells. (A) SICM (left) and TEM (right) images of untransfected Jurkat cells (TEM scale bar is 1 µm). (B) SICM image of VLPs on a cell fixed at 24 hours post transfection. (C) Immunofluorescence staining for tetherin of tetherin-positive (left) and tetherin-negative (right) cells with PE anti-human CD317/tetherin antibody. (D) Immunofluorescence staining of tetherin-negative cells with PE-labelled anti-human CD365/TIM-1 antibody. Images were normalised peak-to-peak, see scale bar for fluorescence intensity values. (E) Images of a Jurkat cell transfected with H2B-mPlum/Gag before (left) and after (right) treatment with protease. (F) Effect of protease treatment on the density of cell membrane bound VLPs. (G) Dimensions of VLPs formed by unlabelled Gag in cells expressing H2BmPlum/Gag, and labelled VLPs in cells expressing 1:1 Gag-GFP:SynGP and 1:1Vpr-GFP:SynGP. Two sample t-test, * $p<0.05$.

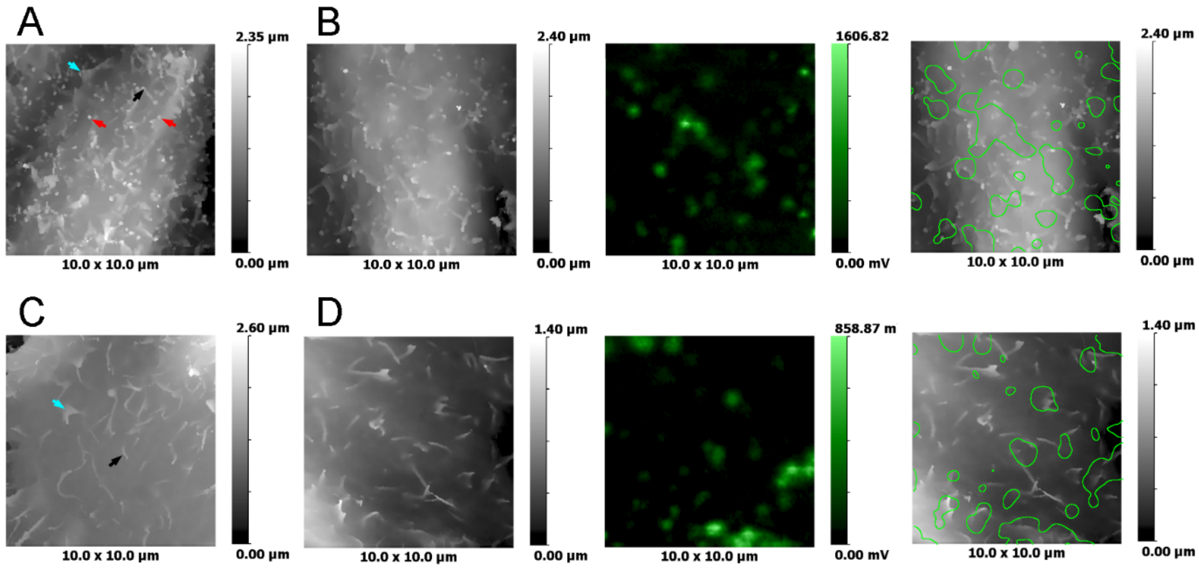

Figure S2. Cell surface structures in HeLa cells. (A and C) Untransfected cells; Stump-like microvilli protrusions (red arrows), finger-like microvilli protrusions (black arrow), and membrane flaps corresponding to dorsal ruffles (cyan arrow). (B and D) Cells transfected with Gag-GFP.

www.pnas.org/cgi/doi/10.1073/pnas.2008156117

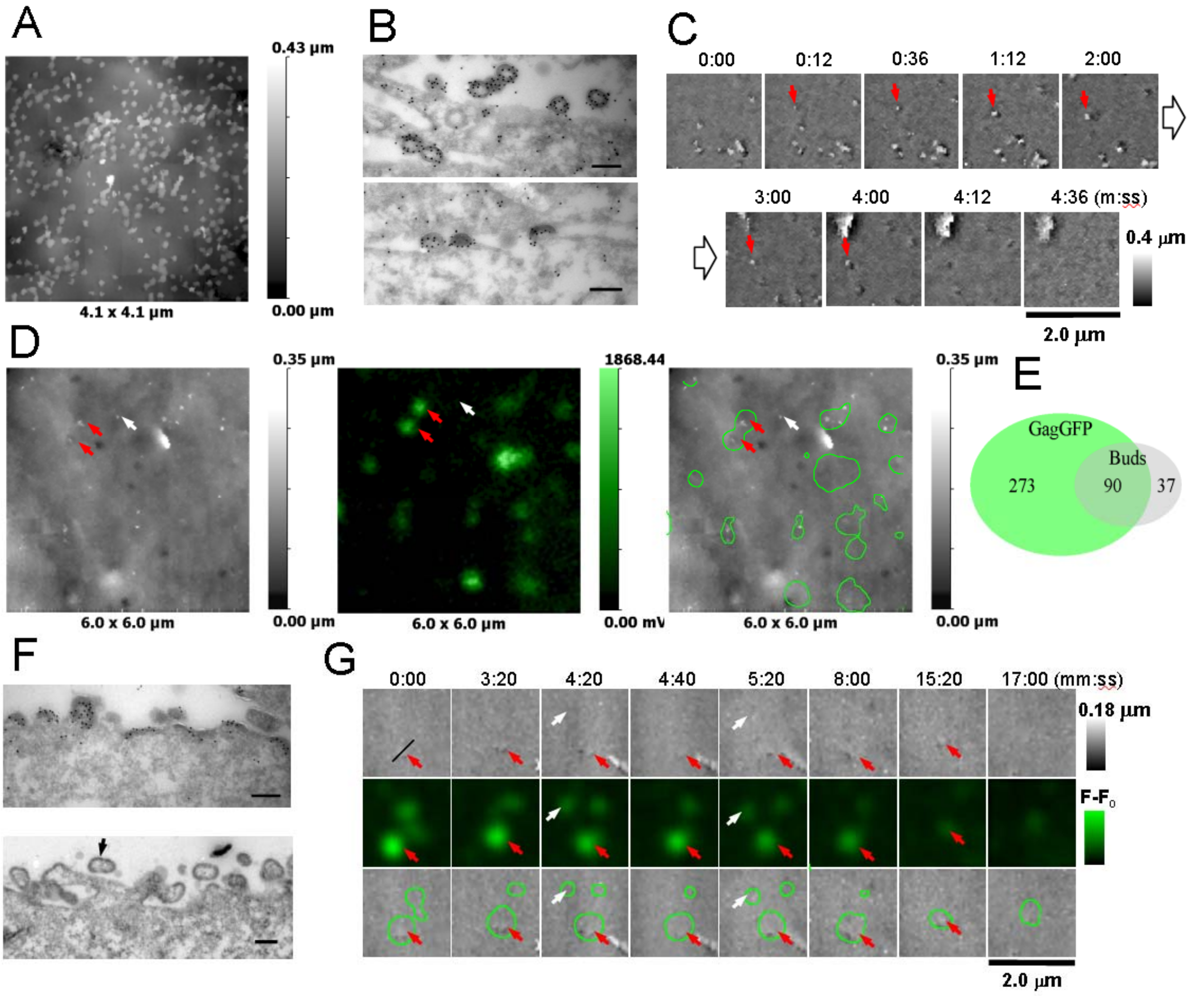

Figure S3. HIV VLP formation in Cos-7 cells. (A) SICM image of VLPs on a cell fixed 26 hours post transfection with H2B-mPlum/Gag. (B) TEM images of Gag VLPs and buds formed in unrestricted membrane areas (top) and cell-cell contacts (bottom) of cells transfected with SynGP. (C) HS-SICM time-lapse images showing VLP formation (red arrows) in cells recorded 26 hours post transfection. Images acquired at 5 frames per minute. Selected frames are shown. (D) Correlative SICM-FCM images of a cell fixed 24 hours post transfected with 1:1 Gag-GFP:SynGP showing examples of VLP buds with and without associated Gag-GFP fluorescence (red and white arrows respectively). (E) Euler diagram showing the relative overlap of Gag-GFP fluorescence spots and topographically detected buds on fixed cells. (F) TEM images of immunolabeled cryosections of Cos7 cells transfected with 1:1 Gag-GFP:SynGP. Black arrow points at a discontinuity in the Gag layer in an aberrantly shaped particle. Rabbit anti-p24 and p17/Protein A-gold is used in TEM images. Scale bars = 200 nm. (G) SICM-FCM time-lapse images showing Gag-GFP fluorescence spots correlating with flat (white arrow) and lightly curved (red arrow) Gag assemblies. Images acquired at 3 frames per minute; selected frames are shown.

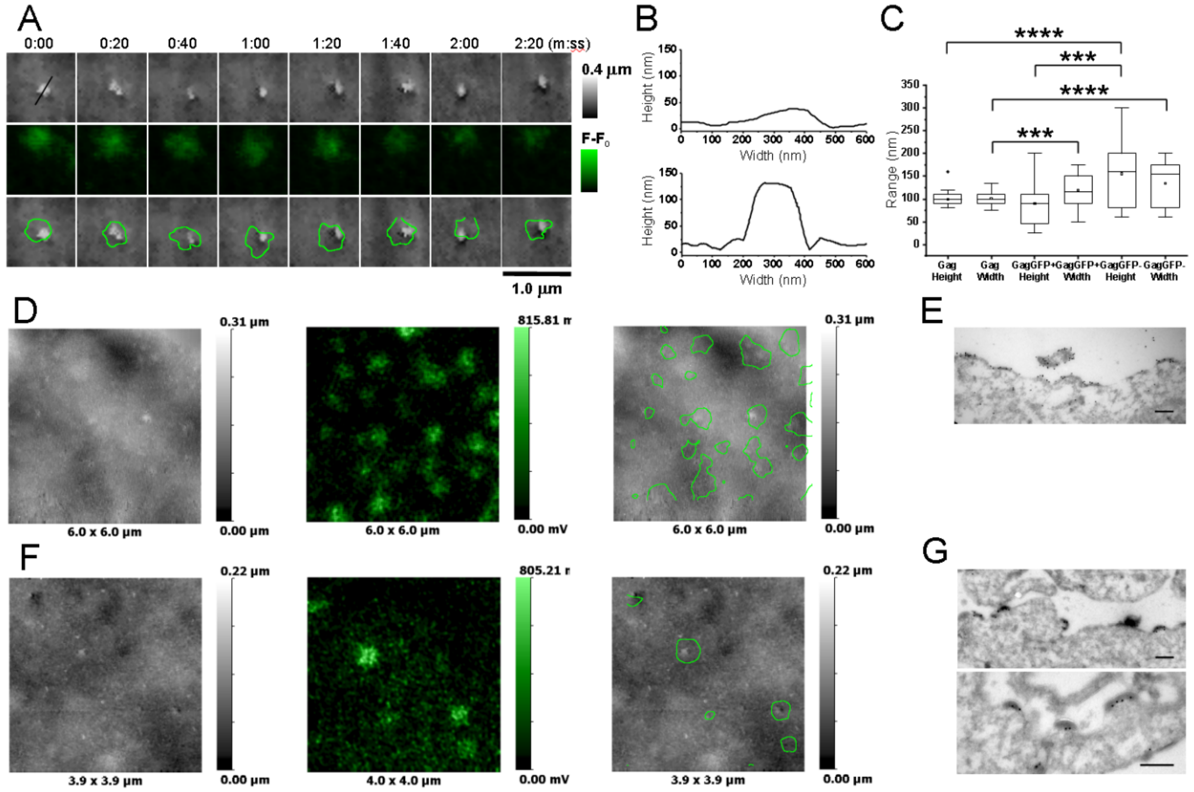

Figure S4. VLP formation in Cos-7 cells. (A) SICM-FCM time-lapse images showing full size VLP and associated Gag-GFP fluorescence in a cell transfected with 1:1 Gag-GFP:SynGP. Images were acquired at 3 frames per minute. (B) Cross section profiles of membrane protrusions corresponding to lightly curved Gag assembly in Fig. 5 F (top) and the VLP in A at 0:00 (bottom). (C) Dimensions of VLPs formed by unlabelled Gag in cells expressing H2BmPlum/Gag, and Gag-GFP positive and negative protrusions in cells expressing 1:1 Gag-GFP:SynGP. Two sample t-test, *** p<0.001, **** p<0.0001. (D) SICM-FCM images of a cell fixed 24 hours post transfection with 1:5 Gag-GFP:SynGP showing flat and lightly-curved buds. (E) TEM image of an immunolabeled cryosection of a cell transfected with Gag-GFP. (F and G) SICM-FCM and TEM images of Cos-7 cells transfected with 1:1 Vpr-GFP:SynGP showing flat and lightly-curved buds. Rabbit anti-p24 and p17/Protein A-gold is used in TEM images. Scale bars = 200 nm.

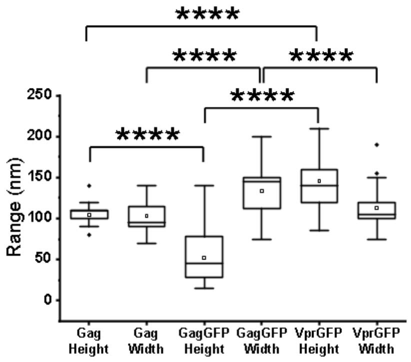

Figure S5. VLP sizes in HEK293T cells. Dimensions of VLPs formed by unlabelled Gag in cells expressing H2BmPlum/Gag, and labelled VLPs in cells expressing 1:5 Gag-GFP:SynGP and 1:1Vpr-GFP:SynGP. Two sample t-test, **** p<0.0001.

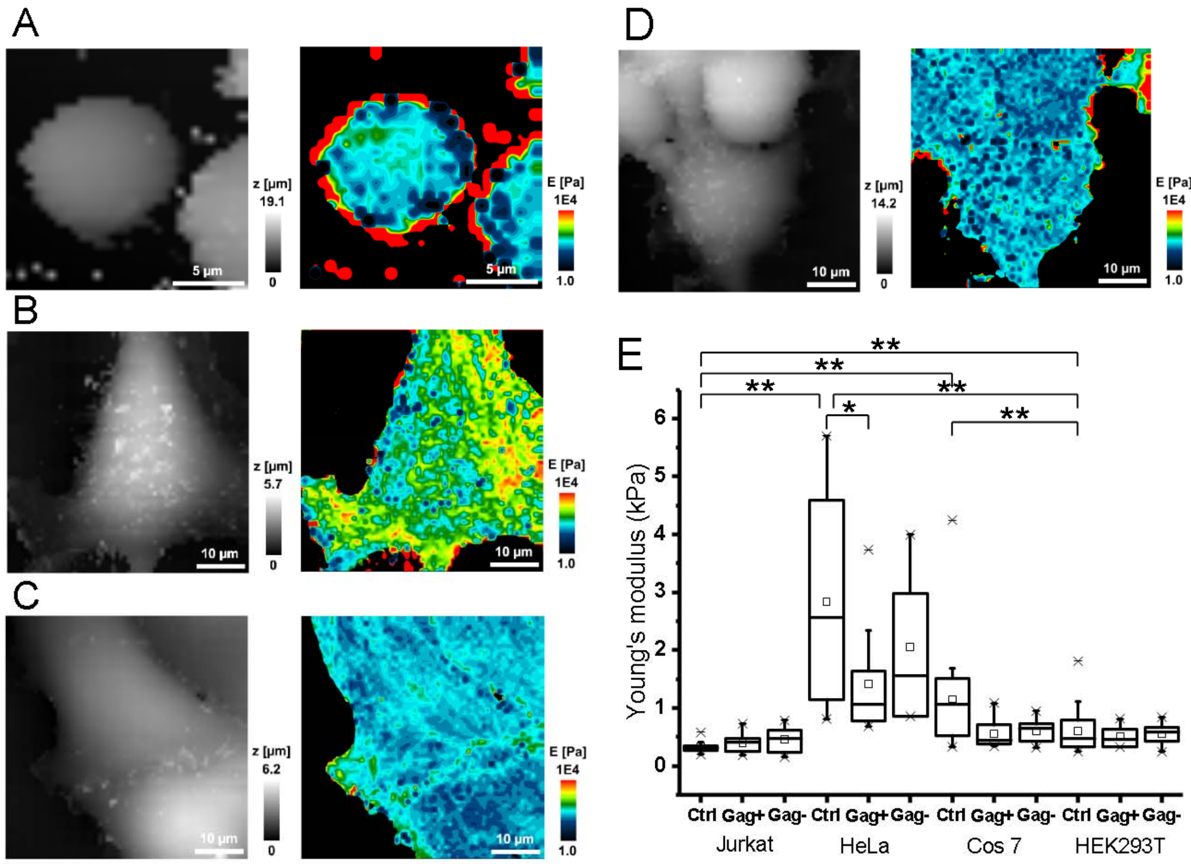

Figure S6. Cell stiffness mapped by SICM. (A-D) SICM topographical images (left) and SICM stiffness maps (right) of Jurkat (A), HeLa (B), Cos-7 (C) and HEK293T (D) cells transfected with H2B-mPlum/Gag. Stiffness of the substrate in the images was set to black colour corresponding to 1 Pa for clarity. (E) Comparison of cell stiffness for control and transfected cells: positive (Gag+) and negative (Gag-) for H2B-mPlum fluorescence. Jurkat (n=10, 13 and 8), HeLa (n=12,10 and 5), Cos-7 (n = 18, 9 and 9) and HEK293T (n=27,9 and 16). Two sample t-test, * $p<0.05$, ** $p<0.01$.

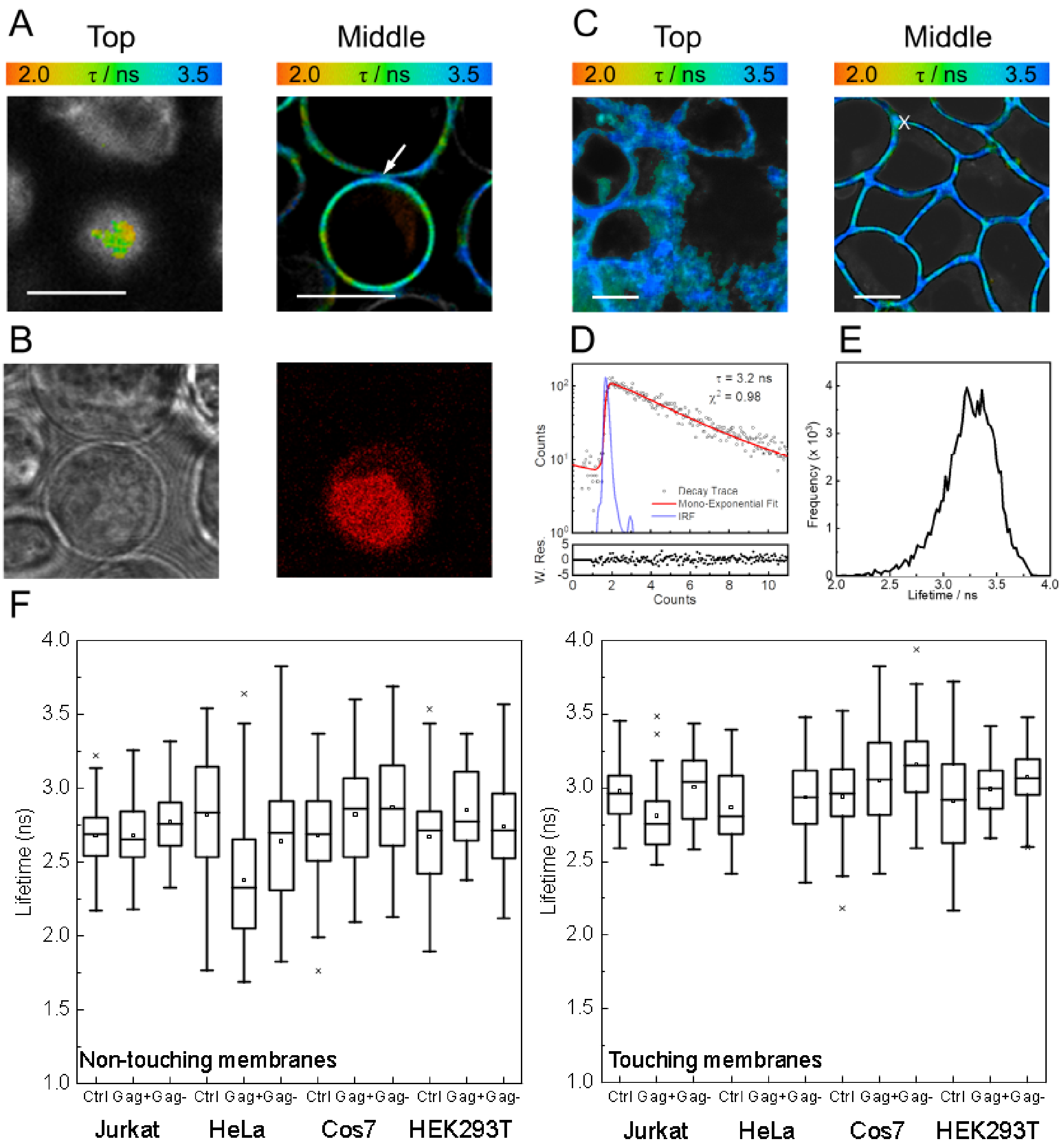

Figure S7. Cell membrane microviscosity measured by Fluorescence Lifetime Imaging Microscopy (FLIM) using BODIPY-C6++ molecular rotor. Higher lifetime indicated by the blue colour in the images corresponds to higher viscosity, according to a prior rotor calibration. (A) FLIM images of Jurkat cells, sectioned at the top (left) and middle i.e. equatorial (right) level. The arrow points at an example of touching membranes. (B) Bright field and fluorescence images showing transfected Jurkat cell measured in A. (C) FLIM images of HEK293T cells, sectioned at the top (left) and equatorial (right) levels. (D) A typical time-resolved fluorescence decay trace taken from image C (middle), from the location shown by the crosshair. The decay is fitted well with a mono-exponential function, verifying the validity of the viscosity analysis; (E) Fluorescence lifetime histogram recorded from image C (middle), the FWHM is 0.5 ns indicating some heterogeneity in membrane viscosities in each recorded image. (F) Summary data for fluorescence lifetime measured in top (left panel) and middle (right panel) membranes in control and transfected cells: positive (Gag+) and negative (Gag-) for H2B-mPlum fluorescence. Scale bars = 10 μM.

Appendix

Materials and Methods

Plasmids.

Gag-GFP was expressed from the plasmid pGag_eGFP, a Rev-independent full-length HIV-1 Gag gene fused to green fluorescent protein (1), kindly provided by Prof. Wesley Sundquist, University of Utah. The plasmid piGag-SNAP was generated from pGag_eGFP by Dr. David Nkwe and Ms. Carina Bannach (LMCB, UCL) by deleting the GFP sequence and inserting an internal SNAP tag (New England Biolabs (UK) Ltd.) between the MA and CA domains of Gag. The codon-optimised synthetic Gag-Pol construct pSynGP (2) was kindly provided by Prof. Stuart Neil, King's College London. Vpr containing an N-terminal GFP tag (3) was kindly provided by Prof. Greg Towers, UCL. The H2B mPlum/Gag plasmid (4) was purchased from Addgene (#102255).

Cell culture and transfection.

Jurkat-TAg cells (kindly provided by Prof. Stuart Neil, King's College London), are a derivative of $CD4^+$ cells that have no detectable cell surface tetherin (5, 6), so that fully assembled VLPs should be released. Cells were cultured in RPMI 1640 (Gibco) supplemented with 10% heat inactivated FBS (Sigma) and 1% glutamax (Gibco) in a 37°C incubator with an atmosphere of 5% CO_2 in air. For microscopic analysis 1.8×10^6 cells/well were seeded in 4-well plates and placed back in the incubator for 5 hours. The cells were then transfected with mix of either Gag-GFP or Vpr-GFP and SynGP or pH2B-mPlum/Gag plasmid DNA using Lipofectamine LTX (Invitrogen), according to the manufacturer's protocol. The total amount of plasmid was always 1.8 µg/well. After transfection, cells were placed in a 37°C incubator with an atmosphere of 16.5% CO_2 in air; this increase in CO_2 concentration in the incubator atmosphere causes decrease of culture medium pH from 7.4 in 5% CO_2 to 7.0 in 16.5% CO_2. This pH has been reported to be optimal for lymphocyte proliferation and reduces apoptosis in cell culture (7, 8). Prior to microscopic analysis, cells were seeded onto poly-l-lysine-coated glass bottom dishes (P35G-0-14-C, MatTek Corporation, USA) and allowed to adhere for 30 min.

HeLa, Cos7 or HEK293T cells were cultured in DMEM (Gibco) supplemented with 10% heat inactivated FCS (Sigma) and 1% glutamax (Gibco) in a 37°C incubator with an atmosphere of 5% CO_2 in air. For microscopic analysis, cells were seeded in glass bottom dishes (P35G-0-14-C, MatTek Corporation, USA) at 50% confluency and incubated for 24 hours. The cells were then transfected with mix of either Gag-GFP or Vpr-GFP and SynGP or with piGag-SNAP only, using either Lipofectamine 2000 (Invitrogen) for Cos7 cells or FuGene 6 (Promega) for HEK293T and HeLa cells, according to the manufacturer's protocols. The total amount of plasmid was always 1.8 µg/dish. Live imaging was performed between 16 and 26 hours after transfection in phenol red free L-15 Medium (Leibovitz) or DMEM (Gibco).

Immunolabeling.

10^6 Jurkat cells were washed with HBSS, 10mM HEPES, 1% glutamax, 5% FBS, pH 6.9. The cells were then incubated for 20 mins on ice in 100µl of the same medium containing 10µg/ml of either anti-human CD317 (BST2/Tetherin; BioLegend) labelled with phycoerythrin (PE) or anti-human CD365-PE (Tim-1; BioLegend). Subsequently, cells were washed twice with ice cold HBSS, 10mM HEPES, 1% glutamax, pH 6.9. For confocal imaging, cells were seeded in glass bottom dishes coated with poly-l-lysine, incubated on ice for 15 min and fixed with 0.5 % formaldehyde.

Subtilisin A protease stripping

Bacillus licheniformis Subtilisin A (Sigma-Aldrich), in lyophilized powder form, was dissolved in HBSS and used at 100µg/mL final concentration. SICM imaging was performed in RPMI supplemented with 1% glutamax in room temperature with an atmosphere of 5% CO_2 in air.

SICM-FCM

Correlative SICM and confocal live imaging was performed between 6 and 26 hours after transfection in HBSS (Gibco) supplemented with 10mM HEPES (Sigma) and 1% glutamax, pH 6.9 or in culture medium. Where specified, cells were fixed in 0.5% formaldehyde in HBSS supplemented with 10mM HEPES and 1% glutamax.

All experiments were performed using a custom built SICM setup consisting of XY-stage (45 x 45 µm, Direct Drive, Capacitive Sensors, Parallel Metrology, IC-XY-4545-001) and Z-stage (25 µm, Direct Metrology, Capacitive Sensor, IC-Z-25-001) powered by Piezo Controller System (IC-PDC-001) and operated using ICAPPIC Controller (IC-UN-001, ICAPPIC Ltd., UK). To allow high speed imaging the setup was equipped with a fast Z piezo actuator (IC-HSZ-001, ICAPPIC Ltd. UK). Fine alignment of the scanning nanopipette tip with the laser beam in the XY plane was done using two N-470 PiezoMike Linear Actuators (Physik Instrumente, Germany) and was based on the optical image acquired using a 100×/1.3NA oil immersion objective. Therefore, partial offset of fluorescence images in relation to topography could be observed.

Nanopipettes were pulled from BF-100-50-7.5 borosilicate glass capillaries (Sutter Instrument Co., USA) using a P-2000 laser puller (Sutter Instrument Co., USA). Ion current was detected by Axopatch 200B amplifier (Molecular Devices, UK) using a gain of 1 mV/pA and a low-pass filter cutoff setting of 5 kHz. The internal holding voltage source of the Axopatch 200B was used to supply a direct current voltage of +200 mV to the pipette. The ion current and outputs of the capacitive sensors from all three piezo elements were monitored using Axon Digidata 1322A and Clampex 9.2 (Molecular Devices, UK).

The excitation was provided by a 488-nm wavelength diode-pumped solid-state laser (Laser 200; Protera). Fluorescence images were recorded using a D-104 Microscope Photometer (Photon Technology International, Inc. USA) through a 100×/1.3NA oil immersion objective.

SICM-FCM control, data acquisition and analysis were performed using custom modified HPICM scanner software and SICM Image Viewer (ICAPPIC Ltd, UK).

Electron microscopy

Correlative light and electron microscopy: Cells expressing mixtures of either Gag-GFP or Vpr-GFP and SynGP were fixed with 3% formaldehyde and 2% sucrose dissolved in HBSS, 10mM HEPES, 1% glutamax for 30 min. and subsequently in 2% formaldehyde and 1.5% glutaraldehyde in HBSS for another 30 min. Cells expressing pH2B-mPlum/Gag were fixed with a mix of 1.5% formaldehyde and 0.1% glutaraldehyde in HBSS, 10mM HEPES, 1% glutamax. Transfected cells were identified by fluorescence imaging and their location on gridded glass bottomed dishes (MatTek Corp, USA) was imaged and documented using an inverted fluorescence widefield microscope (Leica, UK). Aldehyde-fixed cells were then post-fixed for 1 hr at 4°C in 1% OsO4/1.5% K3[Fe(CN)6], and treated with 1.5% tannic acid. Samples were dehydrated in graded ethanol and embedded in Epon 812 (TAAB Laboratories, UK). The transfected cells were then relocated on the resin block surface and sectioned either in xy or xz orientation. Ultrathin sections (~70nm) were cut on an UC7 ultramicrotome (Leica Microsystems, UK), collected on formvar-coated slot grids and stained with lead citrate.

Immuno-electron microscopy: Transfected or untransfected control cells were fixed by adding an equal volume of pre-warmed double-strength fixative (8% PFA in 0.1 M NaPi buffer, pH 7.4) directly into the culture medium. After 10 min, the medium was replaced with 4% PFA and fixation continued for 90 min. Cells were rinsed in PBS containing 25 mM glycine, embedded in gelatine (12%), infiltrated

with 2.3 M sucrose, and frozen in liquid nitrogen. Semi-thin (0.5 µm) or ultrathin (50 - 60 nm) cryosections were cut on an Ultracut UCT microtome equipped with an EM FCS cryochamber (Leica Microsystems, UK). Ultrathin cryosections (50 - 60 nm) on formvar-coated grids were quenched in 50 mM glycine/50 mM NH_4Cl and labelled with primary antibodies and protein A-gold (PAG: The EM Laboratory, Utrecht University, Utrecht, The Netherlands). Sections stained with mouse mAbs were incubated with a rabbit anti–mouse bridging antibody (DakoCytomation, Stockport, UK) before labelling with PAG (5- or 10 nm).

Sections were examined with a Tecnai G2 Spirit transmission EM (Thermo Fisher Scientific, The Netherlands) and digital images were recorded with a Morada CCD camera (Olympus Soft Imaging System, Germany) and the iTEM software package. Images were adjusted for brightness and contrast and figures were assembled using Photoshop CS.

FLIM acquisition and analysis

FLIM was performed through time-correlated single-photon counting (TCSPC), using an inverted confocal laser scanning microscope (Leica, SP5 II) and a SPC-830 single-photon counting card (Becker & Hickl GmbH). A mode-locked femtosecond Ti:Sapphire laser (Coherent, Chameleon Vision II) operated at 930 nm (140 fs pulse duration, 80 MHz) was used as the excitation source with a PMC-100-1 photomultiplier tube (Hamamatsu) detector. Fluorescence emission from BODIPY rotor (500-580 nm) was collected using 700 and 715 nm short-pass filters. A 100x (oil, NA = 1.4) objective was used to collect images at 256 x 256 pixel resolution. Samples on chamber slides were mounted in the microscope at 21 ºC and kept under an atmosphere of 5% CO_2 in air. The SHG signal from urea crystals was used to obtain the instrument response function (IRF). Mono-exponential fitting was performed in SPCImage v7.4 (Becker & Hickl GmbH) using the nonlinear least squares method (NLLSM) and reconvolution algorithm. The χ^2 value and randomness of residuals were used to indicate the goodness of fit to a mono-exponential decay model. A binning parameter of 3 was used to generate a decay count of at least 100 at the peak maximum. 10 pixels in each image were selected from regions of either cell-cell contact or non-touching membranes and pooled together for the subsequent statistical analysis. Confocal images of mPlum fluorescence (λ_{ex} = 514 nm, λ_{em} = 630-790 nm) were recorded at 1024 x 2014 resolution.

Statistical analysis

Statistical analysis was performed using (OriginPro, OriginLab Corp. USA). HIV VLPs sizes are reported as median and interquartile range (IQR) since the distribution of heights values is non-normal. Two sample t-test was used for comparison, with a value of $p<0.05$ considered significant.

Referenses

1. L. Hermida-Matsumoto, M. D. Resh, Localization of Human Immunodeficiency Virus Type 1 Gag and Env at the Plasma Membrane by Confocal Imaging. *J. Virol.* **74**, 8670 (2000).
2. E. Kotsopoulou, V. N. Kim, A. J. Kingsman, S. M. Kingsman, K. A. Mitrophanous, A Rev-independent human immunodeficiency virus type 1 (HIV-1)-based vector that exploits a codon-optimized HIV-1 gag-pol gene. *J. Virol.* **74**, 4839–52 (2000).
3. D. McDonald, *et al.*, Visualization of the intracellular behavior of HIV in living cells. *J. Cell Biol.* **159**, 441–452 (2002).
4. J. Gunzenhäuser, R. Wyss, S. Manley, A Quantitative Approach to Evaluate the Impact of Fluorescent Labeling on Membrane-Bound HIV-Gag Assembly by Titration of Unlabeled Proteins. *PLoS One* **9**, e115095 (2014).
5. E. Erikson, *et al.*, In vivo expression profile of the antiviral restriction factor and tumor-

targeting antigen CD317/BST-2/HM1.24/tetherin in humans. *Proc. Natl. Acad. Sci.* **108**, 13688–13693 (2011).

6. J. Weinelt, S. J. D. Neil, Differential sensitivities of tetherin isoforms to counteraction by primate lentiviruses. *J. Virol.* **88**, 5845–58 (2014).

7. K. S. Carswell, E. T. Papoutsakis, Extracellular pH affects the proliferation of cultured human T cells and their expression of the interleukin-2 receptor. *J. Immunother.* **23**, 669–74.

8. D. A. Loeffler, P. L. Juneau, S. Masserant, Influence of tumour physico-chemical conditions on interleukin-2-stimulated lymphocyte proliferation. *Br. J. Cancer* **66**, 619–22 (1992).
